# Breath Analysis: Comparison among Methodological Approaches for Breath Sampling

**DOI:** 10.3390/molecules25245823

**Published:** 2020-12-10

**Authors:** Alessia Di Gilio, Jolanda Palmisani, Gianrocco Ventrella, Laura Facchini, Annamaria Catino, Niccolò Varesano, Pamela Pizzutilo, Domenico Galetta, Massimo Borelli, Pierluigi Barbieri, Sabina Licen, Gianluigi de Gennaro

**Affiliations:** 1 Department of Biology, University of Bari, 70126 Bari, Italy; ventrella.gianrocco@gmail.com (G.V.); facchini.laura@gmail.com (L.F.); gianluigi.degennaro@uniba.it (G.d.G.); 2 Apulian Breath Analysis Center (CeRBA), Istituto di Ricovero e Cura a Carattere Scientifico (IRCCS), Istituto Tumori “Giovanni Paolo II”, 70124 Bari, Italy; 3 Thoracic Oncology Unit, Istituto di Ricovero e Cura a Carattere Scientifico (IRCCS), Istituto Tumori “Giovanni Paolo II”, 70124 Bari, Italy; a.catino@oncologico.bari.it (A.C.); nicco.varesano@gmail.com (N.V.); pamela.pizzutilo@gmail.com (P.P.); galetta@oncologico.bari.it (D.G.); 4 Department of Chemical and Pharmaceutical Sciences, University of Trieste, 34127 Trieste, Italy; borelli@units.it (M.B.); barbierp@units.it (P.B.); slicen@units.it (S.L.)

**Keywords:** standardization, breath analysis, breath sampling, breath sampling devices, end-tidal breath, ReCIVA, Mistral

## Abstract

Despite promising results obtained in the early diagnosis of several pathologies, breath analysis still remains an unused technique in clinical practice due to the lack of breath sampling standardized procedures able to guarantee a good repeatability and comparability of results. The most diffuse on an international scale breath sampling method uses polymeric bags, but, recently, devices named Mistral and ReCIVA, able to directly concentrate volatile organic compounds (VOCs) onto sorbent tubes, have been developed and launched on the market. In order to explore performances of these new automatic devices with respect to sampling in the polymeric bag and to study the differences in VOCs profile when whole or alveolar breath is collected and when pulmonary wash out with clean air is done, a tailored experimental design was developed. Three different breath sampling approaches were compared: (a) whole breath sampling by means of Tedlar bags, (b) the end-tidal breath collection using the Mistral sampler, and (c) the simultaneous collection of the whole and alveolar breath by using the ReCIVA. The obtained results showed that alveolar fraction of breath was relatively less affected by ambient air (AA) contaminants (*p*-values equal to 0.04 for Mistral and 0.002 for ReCIVA Low) with respect to whole breath (*p*-values equal to 0.97 for ReCIVA Whole). Compared to Tedlar bags, coherent results were obtained by using Mistral while lower VOCs levels were detected for samples (both breath and AA) collected by ReCIVA, likely due to uncorrected and fluctuating flow rates applied by this device. Finally, the analysis of all data also including data obtained by explorative analysis of the unique lung cancer (LC) breath sample showed that a clean air supply might determine a further confounding factor in breath analysis considering that lung wash-out is species-dependent.

## 1. Introduction

In recent decades, volatile organic compounds (VOCs) analysis in the exhaled breath has gained a great interest by the scientific community at a world level due to promising results obtained using this technique for diseases diagnosis and, thanks to its low cost, minimal invasiveness and ease of use. Nevertheless, to date, it still remains an underutilized technique in clinical practice due to the lack of standardized procedures for breath collection and analysis able to guarantee a good reproducibility of the technique and comparability of results. Another critical point for the use of breath analysis in clinical practice is the partial knowledge of which factors play a role in the VOCs’ exhaled breath profile definition. As already known, exhaled breath consists of an inorganic fraction (nitrogen, oxygen, carbon dioxide, and inert gases) and an organic one mainly composed by VOCs as isoprene and acetone and by numerous trace compounds belonging to several chemical classes like alcohols, ketones, aldehydes, terpenes, and aromatics, etc. [1,2,3,4]. These exhaled VOCs can be endogenous and exogenous ones. Endogenous VOCs are produced by metabolic processes that take place in different body districts and, through the blood stream, are transported to the lungs where they spread into the exhaled breath flow by the alveolar gas exchange mechanism. Exogenous VOCs are mainly environmental pollutants that are taken up by the body via inhalation and skin adsorption or species absorbed through food ingestion. On the bases of these considerations, several studies demonstrated that the identification of a specific pattern of endogeneous VOCs’ characteristics of a disease is a useful tool for its early diagnosis [5,6,7]. However, it is important to consider that changes in VOCs’ profile in human breath could be due to: (a) exogenous VOCs linked to ambient air where breath sampling occurs, (b) environmental exposure to VOCs, (c) confounding factors such as sex, diet, genetics, smoking habits, etc., and (d) differences in VOCs’ diffusion from blood to alveolar air depending on their physic-chemical properties such as polarity, volatility, and solubility in fat [8]. All these factors in addition to exogenous VOCs could interfere in the identification of disease markers in breath analysis. In fact, except for the most common molecules of human breath, such as acetone and isoprene, many of the remaining trace-compounds could be related to exogenous origins [3,9]. Therefore, it is mandatory in order to achieve a correct identification of biomarkers molecules, to evaluate which compounds are realistically endogenous [8,9]. Finally, another issue in breath analysis is the variability of the exhaled breath composition depending on breathing depth and sampling modality such as multiple breaths rather than a single breath or alveolar fraction when compared to whole breath [10]. As reported by several studies, even if the endogenous VOCs comes from the blood-gas exchange, their presence in human breath can be affected by normal lung physiology and by hypo or hyper ventilation conditions as well [11,12,13,14,15]. Moreover, it was demonstrated that the gas exchange of highly soluble VOCs such as acetone promptly occurs and they are more present in whole breath rather than in the alveolar fraction, while, for low blood soluble VOCs, the exchange is mainly alveolar [10,16]. In fact, Španěl et al. found isoprene concentration in the alveolar portion three times higher than in the entire exhaled air [17]. In addition, there is evidence that a better reproducibility of data is obtained when only the end-tidal fraction of breath is analyzed, making the end-tidal collection desirable with respect to the mixed breath analyses [18,19]. Nevertheless, the end-tidal phase collection requires more sophisticated devices for breath sampling such as the CO_2_ sensor for CO_2_ level monitoring or the exhaled breath volume control in order to select only the last 200/300 mL of the exhaled breath. Finally, it is mandatory to avoid the contaminations during breath sampling and transport. In fact, to date, the most diffuse off-line breath sampling procedure consists of collection of human breath into polymeric bags and subsequent transfer of VOCs in breath samples into a sorbent tube for thermal desorption analysis coupled with gas chromatography and a mass spectrometry detector (TD-GC/MS). Clearly, this procedure could determine changes in VOC’s profile of the breath sample due to the VOC’s background of bags [20,21,22] and to their storage and transport conditions potentially determining condense formation in the breath sample or VOC’s loss and/or contamination due to permeability of polymeric bags [11,20,23,24,25,26]. Therefore, recently, in order to overcome the limitations related the sample storage and transport, more efficient sampling systems, able to collect whole and/or alveolar breath directly on the sorbent tube, have been developed. Among these, there are systems able to collect only an alveolar fraction of breath by using the continuous monitoring of the CO_2_ partial pressure, such as the system developed by the Loccioni Group HumanCare (Ancona, Italy) [27]. On the other hand, we have developed breath sampling systems, such as the Mistral breath sampler (Predict s.r.l., Bari, Italy), which is able to sample only the end-tidal portion of breath thanks to a volume control system, selecting the last 200 mL of each single breath. In addition, there are also systems, such as the “Respiration Collector for In Vitro Analysis” ReCIVA (Owlstone Medical Ltd., Cambridge, UK) capable of collecting both whole and alveolar portions of the exhaled breath in function of specific measured parameters including the CO_2_ levels. This last device is also equipped with a clean air supply system providing clean air to the volunteers during breath sampling in order to limit breath sample contaminations from exogenous VOCs. However, this approach, based on lung “wash out” and its efficiency, are still under investigation since, as defined by the pharmacokinetic model, the inhaled exogenous species participate in alveoli gas exchange and enter the blood stream according to their blood/air partition coefficients [28]. Consequently, on the basis of their chemical nature, VOCs show different behavior in body uptake, distribution, and elimination, according to their affinity with substrates, such as blood and interstitial fluids, moderately perfused tissues, and poorly perfused tissues, such as adipose and connective tissues [29,30,31].

Although the ReCIVA and Mistral devices represent a significant step toward easy and effective exhaled breath sampling, the cost of these devices is not comparable with exhaled breath bags. Moreover, until now, only two studies focused only on isoprene for ReCIVA [32,33] and none for Mistral were conducted aiming to assess the performances of these devices and their comparability with the most diffuse breath sampling method based on a bag’s use. Therefore, the ambitious aim of this study is to compare, for the first time, three different breath sampling approaches and, more specifically, the whole breath sampling by means Tedlar bags, the end-tidal breath collection using a Mistral sampler, and the simultaneous sampling of the whole and alveolar breath by using ReCIVA. In order to study the differences in the VOCs’ profile when whole or alveolar breath is collected and when pulmonary wash out with clean air is completed.

## 2. Materials and Methods

### 2.1. Experimental Study Design

**Figure 1:**
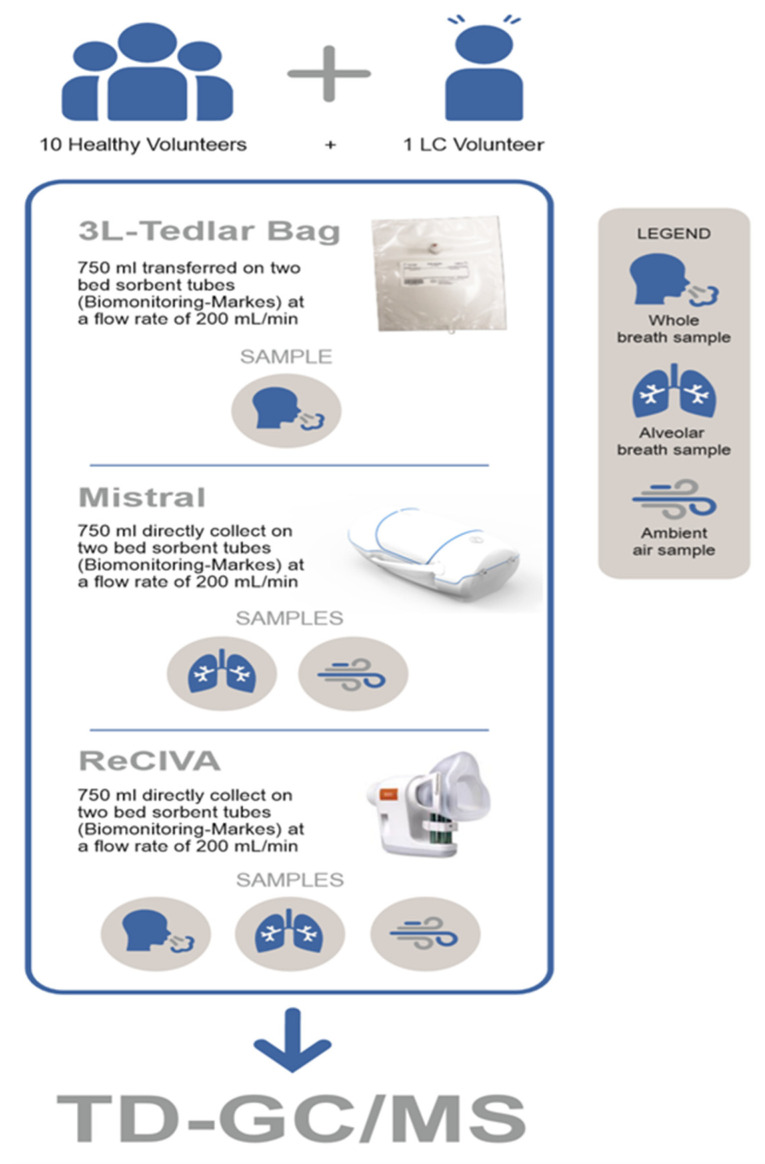
Schematic diagram of the experimental section.

A total of 10 healthy adult volunteers were recruited among medical doctors, nurses, and technicians of the Thoracic Oncology Unit of the IRCCS—Istituto Tumori “Giovanni Paolo II” in Bari, Italy on bases of the studies approved by its Ethical Committee on 14 January 2019 (Prot. No. 679/CE). More specifically, breath samples were collected from five male volunteers with a median age of 40.5 years and from five female volunteers with a median age of 39.8 years. According to specific inclusion criteria, only subjects not affected by upper or lower respiratory tract infections during the last four weeks before the breath sampling and not affected by asthma, chronic obstructive pulmonary disease, or systemic diseases such as diabetes and malignancy, were included in the study. Moreover, in order to minimize the variables that could affect the experimental results, only never-smokers were selected and all volunteers refrained from eating and drinking (except water) for at least 12 h before breath sampling. In fact, samples were collected between 07:00 and 11:00 following an overnight fast. Exhaled breath collection was standardized for all subjects and was carried out in the same room of the Thoracic Oncology Unit where the volunteers remained for at least 10 min before breath collection so that an equilibrium was created between the lung and ambient air. Then, breath samples of each volunteer were sequentially collected: (a) into the Tedlar^®^ bag, (b) by using the Mistral device (Predict s.r.l., Bari, Italy) and, finally, (c) by means of the ReCIVA sampler (Owlstone Medical, Cambridge, UK). Maintaining a normal breathing pattern without restriction or limitation, all volunteers breathed calmly through a mouthpiece (mask for ReCIVA) and slowly exhaled in each of three sampling modalities in order to avoid hyperventilation and bias in the VOCs’ profile [13,14,34]. Among the three different sampling sessions, each volunteer had 10 min of rest. In addition, during each experiment consisting of the collection of breath samples from one volunteer by the three different sampling approaches, two ambient air samples were simultaneously collected by Mistral and ReCIVA samplers. The technical characteristics of different sampling methods are described below and a schematic diagram of the experimental section is shown in Figure 1.

Finally, considering the difficulty for the experimental section and in order to preliminary explore potential differences in the VOCs’ profile due to pulmonary wash-out by synthetic clean air between healthy volunteers and patients, only a breath sample exhaled from one female patient affected by lung cancer was preliminarily collected and analyzed.

### 2.2. Breath Sampling into Tedlar^®^ Bags

All volunteers breathed calmly through a mouthpiece with the nose clipped and slowly exhaled the full expiratory vital capacity. The procedure was repeated two to three times after 2 to 5 min of rest to fill the entire volume of the inert 3L-Tedlar bags L (Restek, Bellefonte, PA, USA). As reported in a previous study [7], a statistical analysis based on several experimental sessions was conducted to evaluate the VOCs’ background of Tedlar bags used in this study and the most effective approach to reduce it. This consisted of filling 10 different bags with wet high-purity grade air and measuring the VOCs’ content over the time and in different cleaning conditions. The VOCs’ background, mainly characterized by Phenol, CS_2_, and *N*,*N*-dimethylacetamide drastically decreased after use. Moreover, memory effects were excluded when bags were flushed three times with high purity grade inert gases and conditioned at a temperature higher than 40 °C. Therefore, in order to guarantee a minimum background, before using, bags were purged three times with ultrapure air (high purity grade, SIAD S.p.A, Bergamo, Italy), then conditioned at 50 °C, and finally flushed with another 3 L of ultrapure air. After breath collection, the breath samples were promptly transferred to sorbent tubes. A total sample volume of 750 mL of exhaled breath were collected on a sorbent tube at a flow rate of 200 mL/min by using a low-flow sampling pump (Pocket Pump, SKC, Houston, TX, USA) and a Teflon transfer line. The flow rate pulled by the pump was verified prior to transferring each breath sample from the bag and for each sorbent tube by using the Gilian Gilibrator-2 system (SENSIDYNE L.P., St. Petersburg, FL, USA). Finally, considering the results reported previously, the VOCs linked to the Tedlar bag background were a priori excluded from the discussion of results of this study and from the comparison among the breath sampling methods.

### 2.3. Breath Sampling by a ‘Mistral’ Device

Mistral is a new breath sampler developed by the medical device retailer and R&D company Predict s.r.l. (Bari, Italy) with the scientific support of the Department of Biology of the University of Bari in the framework of the project entitled “INSiDe the Breath” funded by the Apulia region. Using this device, the breathing follows a natural pattern because mouthpieces and all inner connections and tubes have dimensions able to avoid expiration flow resistance. This device collects exhaled breath using disposable mouthpieces and directly transfers it to a sorbent tube at a selected sample flow rate of 200 mL/min. Thanks to a volume control system and a sampling buffer, it is able to collect only the end-tidal fraction of the exhaled breath and, more specifically, the last 150 mL of exhaled breath. In addition, a thermal control system set to body temperature (ranging from 36 to 37 °C) and a heating plate along sampling lines connecting mouthpieces to sorbent tubes, avoid the formation of condensation. Before each breath sampling session, a device cleaning and ambient air (AA) collection procedures are provided by the system. The cleaning procedure consists of flushing 1 L of air through all device lines while AA collection provides the sampling of 750 mL of room air in the sorbent tube. In this study, each volunteer calmly breathed while seated at rest, until a volume of 750 mL of exhaled end-tidal breath was collected. In order to characterize the VOCs’ background associated with the breath collection system Mistral, 10 experimental sessions were properly scheduled for the assessment of the potential VOC contaminants. More specifically, 10 sorbent tubes were collected by sampling high-purity grade air (SIAD S.p.A) in the same way of breath samples. Detected VOCs were, then, compared with those determined on sorbent tubes obtained by high-purity grade air transferring from Tedlar bags promptly filled. This comparison showed a VOC’s background mainly characterized by 1,4 pentadiene, 2-hexanone, 6-methyl-5-hepten-2-one, and 2,2,4,6,6-pentamethyl-heptane. Likely, these VOCs were emitted by device construction materials. In fact, 1,4 pentadiene is a high-temperature PVC combustion by-product [35], hexanone, and 6-methyl-5-hepten-2-one are volatile components in polyethylene and 2,2,4,6,6-pentamethyl-heptane was found as solubilize for non-hydrocarbon materials [36]. Moreover, the potential memory effect in the collection of breath samples was evaluated in 10 experimental sections by sampling a high-purity grade air sample in the same way as breath sampling after both collection of a real breath sample and a cleaning procedure. The comparisons among the VOCs detected on sorbent tubes collecting high-purity grade air before and after real exhaled breath sample in the different experimental sections showed that cyclopentane and 3,6-dimethyl-decane were found in 70% of cases in an inert gas sample collected after breath sampling. Therefore, VOCs’ contaminants associated with the breath sampling system and those linked with the potential memory effect were excluded from the discussion of results obtained in this study with a method comparing purpose.

### 2.4. Breath Sampling by ‘Respiration Collector for In Vitro Analysis’ (ReCIVA)

The ReCIVA breath sampler is a device designed by Owlstone Medical (Cambridge, UK) to collect two different portions of exhaled breath onto four TD tubes thanks to a continuous monitoring of pressure and CO_2_ levels within a disposable mask during respiration. The device consists of two parallel pumps that are triggered in response to the appropriate phase of the respiratory cycle, aiming the simultaneous collection of two different breath fractions of interest. The standard collection parameter referred to pressure and CO_2_ levels as specified by the manufacturers were used in this study. During the collection of breath, the volunteer breathed tidally while seated at rest and inhaled clean medical air supplied at 40 L/min through the mask, which was sealed to the face so that no ambient air passes through. All ReCIVA masks used in the tests were new and, before breath collection, each mask was baked at 60 °C overnight and then cooled and flushed with clean air. In order to compare the RECIVA and the two breath sample methods described above, pump A has been set to collect the ‘lower airways’ exhaled breath fraction similarly to the Mistral device, while pump B has been set on the collection of the ‘whole breath’ similarly to breath collection into a Tedlar bag. More specifically, according to parameter optimization conducted by Doran et al., for both breath fractions, a breath volume of 750 mL at a sample flow rate of 200 mL/min has been collected [37]. Moreover, Doran et al. assessed VOC contamination from the exhaled breath collection system by connecting the ReCIVA device to a glass head (AMP3 Ltd., Aldershot, UK) and a CASPER air supply [37]. Comparing VOCs collected from the ReCIVA attached to CASPER and glass head and VOCs collected simultaneously from the room air, they found that eight VOCs were contaminants linked to the sampling equipment. More specifically, in addition to cyclopentane, 2-propenamide, and 2,2,4,6,6-pentamethyl-heptane, the other five VOCs detected belonged to the chemical class of siloxanes, which are typical contaminants linked to silicone-based tubing and mask materials. Therefore, on the basis of findings obtained by Doran et al. [37], the previously mentioned VOCs were a priori excluded from the discussion of results obtained in this study with a comparing purpose.

### 2.5. TD-GC-MS Analyses

The breath samples collected by the different approaches previously mentioned were then analyzed by gas-chromatography coupled with a mass spectrometer combined with a thermal desorption (TD-GC/MS). In order to improve the Breath Analysis performance by making the analytical approach more specific for breath samples, the entire methodology, including VOCs sampling and pre-concentration on sorbent tube followed by thermal desorption and determination of VOCs by GC/MS analysis, was optimized with respect to our previous studies [6,38,39]. In particular, specific adsorbents for packing sorbent tubes and a cold trap were chosen in order to better manage wet samples. Moreover, a more specific chromatographic column was chosen and the TD-GC/MS parameters were optimized in order to analyze a wide range of VOCs useful for breath analysis. More specifically, two bed sorbent tubes packed with Tenax TA and Carbograph 5 TD were used to collect exhaled VOCs (Bio-monitoring steel tube, Markes International Ltd., Llantrisant, UK). Tube preconditioning, following each use, was performed with 50 mL·min^−1^ (99.999%) helium flow at 330 °C for 30 min, as recommended by the manufacturer. All tubes were then capped with brass caps and stored at 4 °C until use. A cold trap specific for wet samples (U-T4WMT-2S Water Management, Markes International Ltd., Llantrisant, UK) was used to trap organic compounds between ethane and C20 in a narrow band at the head of the column and a diphenyl dimethyl polysiloxane capillary column was used for specifying VOCs (VOCOL^®^-Supelco). Analysis of VOCs was carried out using a thermal desorber (TD) Unity 2 (Markes International Ltd., Llantrisant, UK) coupled with a gas chromatographer GC-Agilent 7890 and a mass spectrometer MS-Agilent 5975 (Agilent Technologies, Inc., Santa Clara, CA, USA). The operating conditions of analysis are reported in Table 1. Daily response factors and system integrity were evaluated via single-point calibration (Ultra Scientific Cus-23028). The GC-MS chromatograms were analyzed using the GC-MS post-run analysis software (Agilent Mass Hunter Qualitative Analysis-Agilent Technologies Ltd., Santa Clara, CA, USA) integrating only peaks with an intensity five times higher than baseline. Each compound was identified through spectral library matching (Compounds library of the National Institute of Standards and Technology, Gaithersburg, MD 20899-1070 USA) on the bases of the quality of fit with each VOC’s mass spectrum and through comparison with a GC-MS chromatogram obtained by the analysis of VOCs’ standard solution (Ultra Scientific Cus-23028). More specifically, 42 VOCs were identified based on retention times of the compounds included in a customized standard solution covering a wide range of volatility among organic compounds. The other VOCs were identified by the NIST library considering a match score higher than 85% and a peak area was determined by extracted ion chromatogram EIC. In order to highlight the most relevant VOCs that give rise to the most significant differences among the investigated sampling devices, we have explored all peaks in the collected chromatograms even if VOC attributions were not possible and, in addition, the most intense ions for each mass spectrum were considered. A total of 103 species (not considering previously mentioned background contaminants) were considered in statistical treatment.

### 2.6. Data Analysis

Data Analysis was performed by R (v. 3.6.2, The R Foundation, Vienna, Austria)) using tidyverse, car, and companion packages and R Studio (v. 1.2.5033, RStudio Inc., Boston, MA, USA) as an interface. Two-way ANOVA was performed in order to evaluate significant differences between exhaled breath and related ambient air. Two nominal (Sampling mode and Compound) and one measurement variables (peak area) were considered. Residuals normality was checked graphically and data homoscedasticity was tested by Levene’s test for homogeneity of variances. The residuals’ normality assumption ε~ i.i.d. N (0, σ^2^) was met in all experimental sections. In selected cases, a more accurate estimation of homogeneity of variance have been considered by calculating the ratio of the largest treatment variance estimate to the smallest treatment variance estimate. The assumption of homogeneity is satisfied for ratios lower than 3 [40]. The same approach was considered in order to recognize significant differences between breath sampling devices. Two nominal variables (Device and Compound) and one continuous (peak area) have been considered. For all experiments, compounds with significant peak area differences in terms of exhaled breath–ambient air comparison and in terms of devices’ sampling performance have been selected by a one-way ANOVA test, after a preliminary dataset normalization by scaling and centering. For all ANOVA tests, a *p*-value of 0.05 was assumed to be significant. In the second instance, a *p*-value of 0.1 was also taken into account for a deeper data evaluation. Is important to underline that, not knowing data distribution in advance, all data were preliminarily processed by both parametric as ANOVA and a non-parametric test as the Wilcoxon and Kuskal-Wallis tests. Coherent and consistent results were obtained and are reported in the following section.

## 3. Results

A statistical analysis was performed on the data set collected during the 10 different experimental sections (described above) and consisting of two nominal variables: the three different breath sampling approaches and the list of 103 detected VOCs. The data analysis aimed to identify:

- endogeneous and exogeneous VOCs and the ambient air contribution to VOCs’ profiles determined by collecting both whole breath and the alveolar fraction of breath in the presence and in the absence of a clean air supply system (Test 1),
- differences in VOCs’ profiles linked to the collection of lower airways expiratory breath samples and whole expiratory breath samples (Test 2),
- differences in VOCs’ profiles determined by collecting breath samples using the different investigated devices (Test 3).

### 3.1. Test 1: Ambient Air Contribution to Breath Sampling

It is known that contamination from the ambient air is an important issue in exhaled breath collection [17,41,42,43]. In order to deepen this point, a comparison between ambient air and breath samples collected by each of the three different breath sampling approaches was assessed. More specifically, after checking the normality of data by visualizing directly the residuals’ distribution curve and their homoscedasticity by the Levene test, a two-way ANOVA was applied to a data related peak area for each VOC detected in the breath exhaled from 10 healthy volunteers and in ambient air (AA) samples simultaneously collected. The VOCs’ levels detected in the alveolar fraction of breath collected by Mistral and ReCIVA Low resulted in a significant difference at 95% of significance with respect to those detected in ambient air samples with a *p*-values equal to 0.04 and 0.002, respectively. The same result was obtained for Tedlar bags even if, in this case, the comparison has been done with the AA sample collected by ReCIVA (*p*-value equal to 0.0001) and Mistral (*p*-value equal to 0.002) devices. On the contrary, no significant difference was determined between AA and whole breath samples collected by ReCIVA Whole (*p*-value equal to 0.97). More specifically, considering *p*-values obtained for each VOCs, the 55%, 77%, 33%, and 47% of VOCs in breath samples collected by Mistral, ReCIVA Low, ReCIVA Whole, and Bag, respectively, resulted in a significant (at 95% of significance) difference from those in ambient air samples.

**Figure 2:**
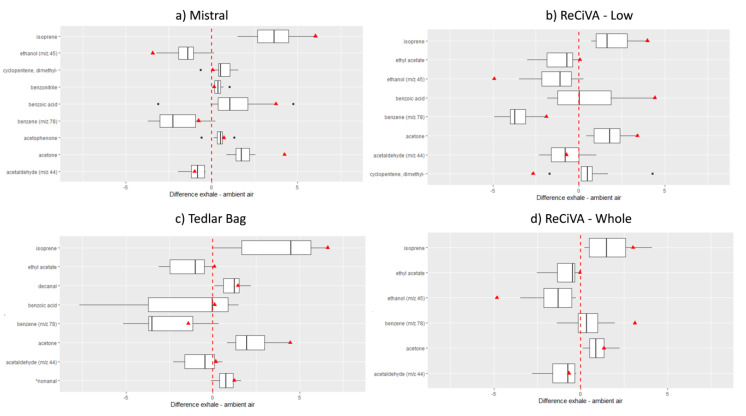
Differences between the normalized peak area of VOCs significantly different at 95% and (*) 90% of significance, respectively, in exhaled breath samples when compared to ambient air samples collected by using Mistral (**a**), ReCIVA Low (**b**), Tedlar bags (**c**), and ReCIVA Whole (**d**). The Red Triangle represents the result related to the only one breath sample collected from a patient affected by Lung Cancer (LC). Black dots represent outliers.

Therefore, a data-related peak area for each VOC detected in breath and ambient air (AA) samples were normalized and their difference were analyzed by one-way ANOVA in order to evaluate the VOCs showing more relevant differences among those having statistically significant differences (at 95% and 90% of significant) between AA and breath samples from healthy volunteers for each investigated breath sampling approach. For example, in Figure 2, the first classified VOCs showing the most significant differences are reported. All data related to the values of mean, median, and standard deviation and the *p*-values obtained by the Wilcoxon test for each VOC found in ambient air and breath samples are reported in Supplementary Materials (Tables S1 and S2). 

As expected, endogenous VOCs such as isoprene and acetone were the most abundant VOCs in lower airways and whole expiration breath samples collected by using all breath sampling approaches (Figure 2a–d) [10,15,17]. Benzoic acid and dimethyl-cyclopentene were more abundant in lower airways’ expiration breath samples collected by using Mistral (Figure 2a) and ReCIVA Low (Figure 2b) with respect to ambient air samples, suggesting an agreement between two breath sampling equipment. Moreover, acetophenone and benzonitrile in the alveolar fraction of exhaled breath collected by Mistral was significantly higher than those measured in room ambient air. On the other hand, as expected, benzene, ethanol, and acetaldehyde in ambient air samples resulted in significantly higher values with respect to their levels in the alveolar fraction of breath collected by using Mistral and ReCIVA Low (Figure 2a,b and Table S2). The same results were obtained for ethyl acetate detected in the alveolar breath samples collected by ReCIVA Low (Figure 2b) and in whole expiration breath samples collected by using Tedlar bags and ReCIVA Whole (Figure 2c,d). An exception was benzene detected into the samples collected by using the ReCIVA Whole approach, resulting in some whole breath samples higher than benzene levels in ambient air room temperature. This finding suggested the ambient air contamination of samples collected using ReCIVA Whole and, thus, the probable ineffectiveness of a clean air supply. In addition, whole expiration breath samples collected by Tedlar bags are also characterized by higher levels of decanal and nonanal values with respect to those detected in ambient air samples at 95% and 90% of significance, respectively (Figure 2c). As specified above, this comparison could be less reliable because AA samples were not collected directly in Tedlar bags but by using Mistral and ReCIVA devices.

Considering alveolar gradient values calculated as (Peak Area (alv) − Peak Area (AA))/Peak Area (alv) and reported in Supplementary Materials (Table S3), a rough differentiation between inhaled and exhaled substances was provided [44]. More specifically, according to results reported by Philips et al. [44] and Miekisch et al. [19], ratio values equal to about 1 were obtained for blood-borne substances of clearly endogenous origin like acetone, isoprene, and dimethyl sulphide. On the contrary, a negative alveolar gradient was obtained for typical exogeneous VOCs as butanal, 2-butanone, toluene, benzene, cyclohexane, etc. In order to enlarge the knowledge reported by Philips et al. [44] and Miekisch et al. [19] and relate it to the normalized alveolar gradient of VOCs in breath, data obtained in this study for all 103 VOCs are reported in Supplementary Materials (Table S3).

### 3.2. Test 2: Fraction of the Breath Sampled

Considering all 103 detected VOCs, the comparison between lower airways’ expiratory breath samples and whole expiratory breath samples collected from 10 healthy volunteers by using Mistral and Tedlar bags, respectively, showed no significant differences in detected VOCs’ profiles (*p*-value equal to 0.7 for all 103 detected VOCs). On the contrary, the comparison between ReCIVA low and ReCIVA Whole showed a significant difference at 95% of confidence (*p*-value equal to 0.006 for all 103 detected VOCs). Data related to the comparison for each VOC are reported in Supplementary Materials Tables S4 and S5. Considering each VOC and comparing results obtained using the Tedlar bag and Mistral, species such as toluene, benzene, propanol, ethanol, and 2-butanone showed ratio values between Mistral and bag even lower than 1, according to data obtained by Salvo et al. [45] and Miekisch et al. [19]. VOCs as toluene and benzene are species characterized by low water solubility and, due to their exogenous nature, had similar levels both in alveolar and mixed breath as for toluene and higher levels in the mixed expiratory phase as for benzene [45]. VOCs, which are assumed exogenous but may be stored in different body compartments like benzene halogen derivate or substances generated in different compartments (e.g., upper airways and the lungs) like dimethylsulfide, showed ratio values even higher than 1 [46,47]. Species such as ethanol was expected to show higher concentration in alveolar breath with respect to a mixed one. Anyway, in this study, ethanol levels in the bag were higher than those determined by using Mistral (alveolar fraction of breath) likely due to an additional contribution from bacterial activity in the mouth and from AA [45]. Despite their endogenous nature, acetone and isoprene showed two relatively different behaviors. In fact, according to Shubert at al. [48], acetone showed values of alveolar to a mixed ratio equal to about 1 while slightly higher values were found for isoprene (mean ratio equal to 1.2). These findings are reasonable considering that acetone, as opposed to isoprene, is a highly soluble volatile and gas exchange occurs in the airways rather than alveoli. The values of alveolar to a mixed ratio for isoprene obtained by comparing levels detected in the bag and Mistral samples were lower than those found in other studies (>1.5) [10,17]. These differences could be explained considering that alveolar and mixed breath were not sampled simultaneously and with the same sampling modality and that, as reported by Herbig et al., [14], isoprene due to its chemical physical proprieties and to its kinetic of the blood–air exchange in the lung, shows higher variability with respect to other VOCs with a relative standard deviation (RSD) of 30% for the same sampling section. Possible significant values in breath sampling by using Mistral could be not excluded so that, further investigations aimed to compare Mistral with reference systems able to collect the alveolar fraction of breath thanks to the CO_2_ control, are mandatory.

Comparable results relating alveolar to mixed ratios were obtained considering ReCIVA Low and whole samples, except for acetone and ethanol showing mean ratios equal to 1.4 and 1.5, respectively. Likely, a clean air supply used by ReCIVA allows us to eliminate an additional contribution linked to ambient air contaminants or species in anatomical dead space, especially when more volatile VOCs occurred. 

**Figure 3:**
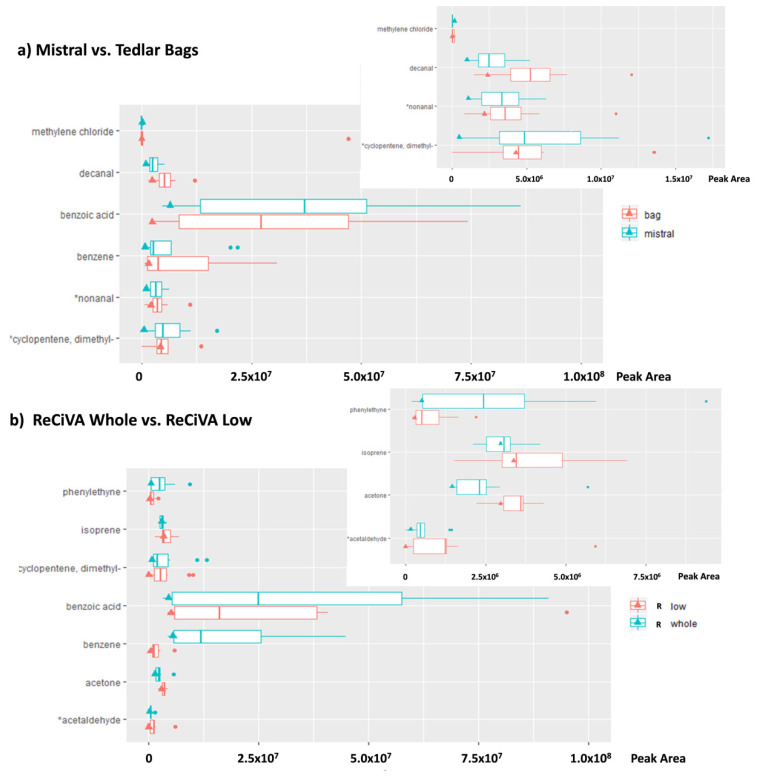
Peak area of volatile organic compounds (VOCs) significantly different at the 95% and (*) 90% of significance, respectively, in lower airways expiratory breath compared to whole expiratory breath. Comparison between Mistral and Bag (**a** and insert) and between ReCIVA Whole and Low (**b** and insert). Triangle represents the peak area value for the LC patient. A zoom of Figure 3a,b on a reduced scale of the *x*-axis is reported in the inserts.

Afterward, for each VOC, a pairwise comparison between Mistral and Tedlar bag data was provided and the resulting differences were analyzed by one-way ANOVA in order to evaluate the VOCs showing the more significant differences (at 95% and 90% of significant). Only couples with significant differences were taken into account in Figure 3. Among the 103 VOCs detected, five compounds with a *p*-value lower than 0.05 and two compounds with a *p*-value lower than 0.1 were found significantly different between Mistral and Tedlar bags. In the same way, only five compounds with *p*-value lower than 0.05 and one compound with *p*-value lower than 0.1 were found significantly different between ReCIVA Low and Whole. More specifically, benzoic acid and dimethyl-cyclopentene were significantly higher in lower airways collected by Mistral while decanal, nonanal, methylene chloride, and benzene were higher in whole breath collected by using Tedlar bags likely due to the higher impact of AA contaminants on whole breath with respect to an alveolar fraction (Figure 3a and insert). Regarding ReCIVA benzoic acid, phenylethine and benzene were significantly higher in whole breath with respect to lower airways as opposed to isoprene, acetone, dimethyl-cyclopentene, and acetaldehyde (Figure 2b and insert). Controversial results were obtained for benzoic acid showing high levels in alveolar fraction of breath collected by Mistral with respect to the bag and in whole breath with respect to an alveolar fraction of breath collected by ReCIVA. Benzoic acid has a controversial nature in breath because it is present in fruits, vegetables, or food preservatives and, thus, it is assumed with the diet and excreted as hyppurate by urine within 1–2 h of injection [49,50]. Therefore, it could be considered an endogenous VOC confirming results obtained by Mistral. On the other hand, considering that volunteers recruited in this study refrained from eating and drinking (except water) for at least 12 h before breath sampling, lower levels of benzoic acid in the alveolar fraction of breath could be expected. A comparison between alveolar and whole breath samples collected by ReCIVA was expected to be more accurate considering that samples were collected simultaneously by the same instrument and using a medical air supply for pulmonary wash-out. Therefore, possible AA contaminations should be excluded and opposite results were expected by using ReCIVA. Therefore, the higher level of benzoic acid in whole with respect to an alveolar fraction of breath confirmed that the medical air supply fails in reducing the contribution of some ambient air contaminants and its effect is species-dependent.

### 3.3. Test 3: Comparison Among Breath Sampling Devices

Starting from the same dataset and the same statistical criteria of previous tests, the third comparison aims putting in evidence significant sampling performance differences among the investigated breath sampling devices: Mistral, ReCIVA, and Tedlar bags. The ANOVA factors such as “Device” (Mistral, Owlstone low, Owlstone whole, and Bag) and “Compound” (103 VOCs) were coupled to 10 observations (healthy volunteers). More specifically, the comparison between the breath sampling approaches based on sampling of the alveolar fraction of breath such as Mistral and ReCIVA Low, resulted in a *p*-value equal to 1.1 × 10^−5^. In the same way, the *p*-value obtained by comparing the two methods for whole expiratory breath sampling such as Tedlar bags and ReCIVA Whole was equal to 5.2 × 10^−4^. These findings suggested a significant difference in the VOCs profile determined by the different breath sampling devices. 

**Figure 4:**
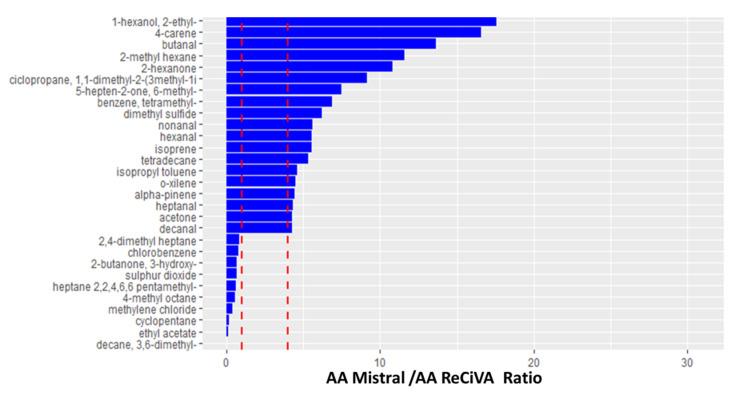
Ratio values between a normalized peak area of VOCs significantly different at a 95% of significance, in Ambient Air samples collected by using Mistral and ReCIVA devices. Red dashed lines represent a ratio value equal to 1 and 4, respectively.

Moreover, the absolute value of peak area of the main investigated VOCs in exhaled breath collected by means Mistral and Tedlar bags resulted in a significantly (at 95% of significance) higher value than those determined in the ReCIVA breath samples. The value of the ratio between the peak area of VOCs sampled by using Mistral and/or bags and ReCIVA resulted in higher values than 1 (ranging from 1 to 25) except for any species as benzene and its halogen derivates, ethyl acetate, cyclopentane, and phenylethine. As reported in Figure 4 and in Supplementary Materials (Table S3), the same differences in VOCs’ levels between Mistral and ReCIVA were found for ambient air samples simultaneously collected. In fact, the values of ratios between the normalized peak area of most VOCs detected in ambient air samples (AA) collected by using Mistral and ReCIVA ranged from 1 to 17.

**Figure 5:**
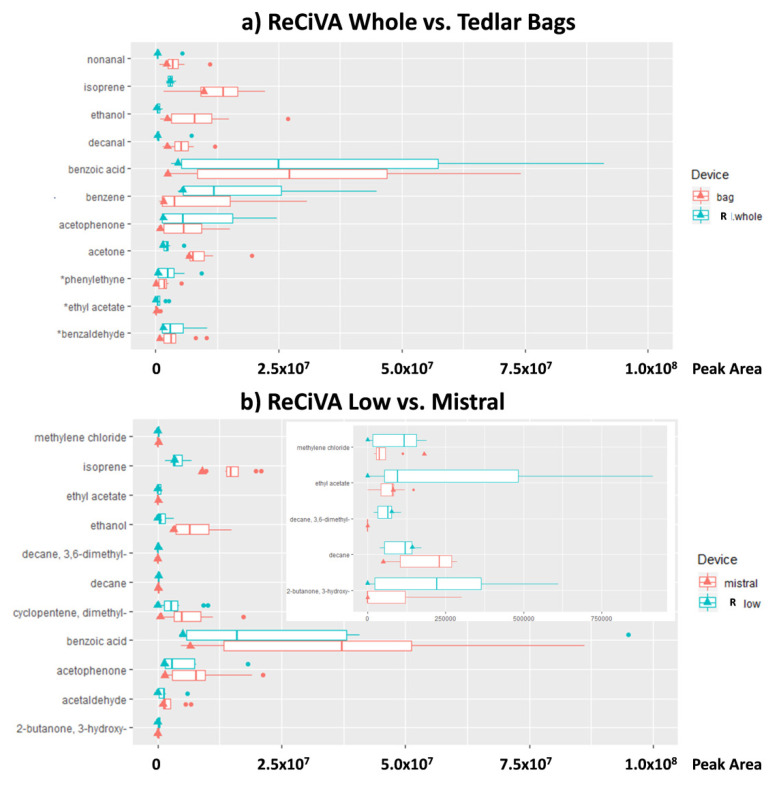
Comparison between a normalized peak area of VOCs significantly different at the 95% and 90% of significance, respectively, in an alveolar fraction of exhaled breath samples collected by using a Mistral and ReCIVA Low approach (**a**) and in whole expiratory breath samples collected by using Tedlar bags and a ReCIVA Whole approach (**b**). The triangle represents a result related to the only one breath sample collected from a patient affected by lung cancer (LC).

Therefore, in order to evaluate the VOCs showing the more significant differences (at 95% and 90% of significant), for each VOC, a pairwise comparison was provided between two couples of breath sampling devices: Mistral versus ReCIVA Low and Tedlar bags versus ReCIVA Whole. The resulting differences were analyzed by one-way ANOVA and only couples with the most significant differences were shown in Figure 5a,b. Data related all detected VOCs that are reported in Supplementary Materials (Tables S1–S3). Eight VOCs were present in significantly different concentrations in whole expiratory breath collected by a Tedlar bag and a ReCIVA Whole approach with a *p*-value lower than 0.05 and three VOCs with a *p*-value lower than 0.1. Among these VOCs, nonanal, isoprene, ethanol, decanal, and acetone in bags were significantly higher than those detected by a ReCIVA Whole approach, while benzoic acid, benzene, acetophenone, benzaldehyde, ethyl acetate, and phenylethyne in bags were significantly lower than those detected by ReCIVA (Figure 5a). Taking into account the comparison between Mistral and ReCIVA Low, eight VOCs resulted in a significant difference at the 95% significance level. Except ethyl acetate, 3-hydroxy-2-butanone, 3,6-dimethyldecane, and methyl chloride, the other VOCs reported in Figure 5b resulted in significantly higher values in breath samples collected by using Mistral with respect to the levels detected in lower airways’ expiratory breath samples collected by using the ReCIVA Low approach.

## 4. Discussion

As expected, the comparison between ambient air and breath samples simultaneously collected by each of the three different breath sampling approaches showed that the alveolar fraction of breath was relatively less affected by AA contaminants with respect to whole breath, even when only healthy volunteers were considered [11,13,19,44,48,51]. In fact, significant differences between VOCs’ profiles determined for AA and alveolar breath samples were obtained for both Mistral (*p*-value equal to 0.04) and ReCIVA (*p*-value equal to 0.002) devices. More specifically, considering *p*-values obtained for each VOCs, the 55%, 77%, 33%, and 47% of all 103 VOCs detected in breath samples collected by Mistral, ReCIVA Low, ReCIVA Whole, and Bag, respectively, resulted in a significant (at 95% of significance) difference from those in ambient air samples.

According to results reported in other papers [13,19], in this study, benzene, toluene, 2-butanone, and butanale showed a negative alveolar gradient resulting in exogenous VOCs while isoprene and acetone, typical endogenous VOCs, in addition to dimethyl sulphide and 1-propanol, showed a positive alveolar gradient. Collected data and results obtained in this study (Supplementary Materials Tables S1–S3) allowed use to expand the database of VOCs showing a positive and negative alveolar gradient than that reported by Philips [44].

Although a greater number of VOCs resulted in a significant difference between alveolar breath and AA samples collected by ReCIVA Low (79 VOCs) with respect to Mistral (55 VOCs), the different values of common VOCs were comparable for both breath sampling devices using or not a clean air supply. Moreover, exogenous VOCs as benzene in whole breath samples collected by using ReCIVA were higher than in the ambient air room, suggesting that lung wash out could determine no significant advantage in a breath sampling standardized procedure. In fact, even if a specific scrubber or a clean air supply could minimize the effect of environmental contamination, the kinetic of each VOC in terms of absorption, metabolism, half-life, diffusion, blood: breath concentrations, and changes could make these “cleaning” approaches ineffective [19,52,53]. For example, lipophilic or less blood-soluble compounds could have longer wash-out rates in the body because they are retained in fat deposits and excreted via breath for a longer time than hydrophilic compounds. In the same way, VOCs from long-term environmental exposure could be up taken into the fatty tissues of the body and they might be slowly and constantly released into the breath [54,55]. Therefore, lung wash out by using scrubber or a clean air supply being specie-dependent, it might induce a further variability factor, which might lead to incorrect data interpretation and, thus, to incorrect breath analysis outputs. Due to further confirmation of the hypothesis, similar results were obtained between the approaches based on use of clean air supply and not when alveolar and mixed breath samples were compared. In Test 2, differences in VOC levels in alveolar and mixed breath samples were found strictly linked to chemical-physical proprieties of compounds and, in particular, linked to their water affinity, solubility, and blood/air partition coefficients instead of pulmonary wash out [19,45,56,57,58]. Moreover, considering the comparison between ReCIVA Whole and Tedlar bags (Figure 5a) endogenous VOCs such as isoprene and acetone resulted in higher values in bags than in breath samples collected by ReCIVA in contrast to likely exogenous VOCs such as benzene and benzoic acid. The opposite results were expected considering the use of a clean air supply in ReCIVA breath sampling. On the basis of these findings, it is reasonable to confirm the previously reported hypothesis about the clean air supply system. In fact, it could affect the breath collection acting in a different way on different VOCs in breath and introducing a further confounding factor in breath analysis. 

Finally, the comparison between the different breath sampling approaches showed that the sampling of the alveolar fraction of breath resulted in a significant difference at a 95% of confidence using Mistral and ReCIVA Low (*p*-value equal to 1.1 × 10^−5^) as well as the sampling of whole breath by using Tedlar bags and ReCIVA Whole (*p*-value equal to 0.0005). These findings suggested a significant difference in human breath VOC profiles determined by using the different breath sampling devices. Opposite to coherent results obtained by using sampling approaches as Mistral and Tedlar bags, the absolute values of peak area of the main investigated VOCs in exhaled breath samples collected by both sampling modalities resulted in higher results than those detected by ReCIVA breath samples. Taking into account that the values of ratios between a normalized peak area of the most of VOCs detected in ambient air samples (AA) collected by using Mistral were even more than four times higher than those determined in AA samples simultaneously collected by ReCIVA (Figure 4), further investigations are needed to deepen the sampling performances and technical characteristics of ReCIVA. In fact, these results confirm the preliminary ones obtained by Harshaman et al. for the isoprene comparing ReCIVA with Altef Bag and two different ReCIVA devices [32,33]. Although the previously mentioned authors recommended an impractical “in-house” flow calibration for each TD tube type and each ReCIVA bank used for collection, in our opinion, a better and online flow mass control should be installed in a ReCIVA device in order to correct the variability in real time associated with the flow impedance through TD tubes. Moreover, although Harshaman et al. [33] in their subsequent study found that the data related to isoprene concentrations resulted in comparable values between two different ReCIVA units, guaranteeing the comparability of results obtained by ReCIVA until now. Further investigations should be performed in order to evaluate their comparability while both considering a wide range of VOCs and with the other studies on breath analysis conducted so far, using bags. On the other hand, despite their endogenous nature, acetone and isoprene detected in this study using bag and Mistral showed values of alveolar to a mixed ratio equal to about 1 and 1.2, respectively, and, thus, lower values (>1.5) reported in other papers [10,11,17,19]. Although these results are reasonable, according to the previously reported considerations, possible significances in breath sampling by using Mistral and ReCIVA could be not excluded so that further investigations, aiming to compare Mistral with reference systems able to collect the alveolar fraction of breath thanks to the CO_2_ control [13,19,48], are mandatory.

Finally, taking into account the difficulty in recruitment of lung cancer (LC) patients able to participate in this experimentation requiring significant physical efforts to provide several breath samples in a unique experimental section. To date, only one breath sample from the LC patient was collected, according to experimental study design. Although we are aware that this breath sample could be not representative of LC patients and the comparison could be piecemeal, we have preliminarily explored the potential differences in VOCs’ profile detected in breath samples from healthy volunteers and LC patients collected by using the different investigated sampling approaches (Supplementary Material Table S6). The levels of the VOCs detected in the unique breath sample collected from the LC patient (triangle in Figure 3a,b and Figure 5a,b) fell in the same quartile with respect to data distribution for both the alveolar fraction and whole breath, confirming no significant differences between the two breath sampling modalities and sampling approaches. Among VOCs such as isoprene, acetone, and benzoic acid in an alveolar fraction of breath as well as benzene in both alveolar fraction and whole breath (Figure 2a–d) shown for the LC sample, the values of differences between a normalized peak area detected in exhaled breath samples compared to ambient air samples with respect to the statistical distribution of other data related to healthy volunteers (HC). On the contrary, these difference values for dimethyl-cyclopentene and ethanol in an alveolar fraction of breath samples collected from the LC patient resulted in lower values than those detected in other HC breath samples. However, it is important to underline that, even if the absolute values of peak area of VOCs as benzene or benzoic acid in the LC breath sample were lower than those determined for HC breath samples (Figure 3). The values related to the difference between the LC breath sample and ambient air sample reported in Figure 2 for these VOCs resulted in higher values than the other data collected from HC breath samples, confirming results obtained in other studies that found these species linked to LC [1,59,60,61,62,63,64,65,66]. In fact, regarding benzene, for example, several studies focused on breath analysis have demonstrated that this exogenous pollutant usually linked to exposure of tobacco smoke and air pollution, could be possible diagnostic biomarkers of cancer able to discriminate between cancer patients and healthy controls [1,6,7,59,60,61,62,63,64,65,66]. It is reasonable considering that patients affected by cancer have been exposed to excessive smoking and/or have experienced continuous occupational exposure by up taking these compounds into the fatty tissues of the body and releasing them into the breath slowly and constantly [63]. Regarding benzoic acid, animal experiments have shown that urinary hippurate excretion as a consequence of benzoate metabolism, is modulated according to the composition of the intestinal microbiome [67,68] and, thus, several diseases such as cancer by altering the gut microbiome, determine a reduction of the hyppurate excreted with urine and an increase of benzoic acid released with breath [67]. Although these findings are preliminary, they are coherent with results obtained in previous studies and suggest the importance to consider the ambient air contribution in each breath sampling section and, thus, the differences between VOCs levels in breath samples and AA instead of absolute value of peak area of each VOCs. In fact, changes in the difference between VOC levels in breath and ambient air samples collected simultaneously, could be a more eloquent factor to consider in breath analysis for early diagnosis.

## 5. Conclusions

Although breath analysis results are interesting and open up a fascinating scenario in cancer research applications, to date, breath analysis is not yet used in clinical practice due to the lack of standardized analytical methods and to the method-dependent variability associated with analysis output. It is well known that the VOCs levels in human breath samples are strongly affected by sampling modality and parameters used for exhaled breath samples’ collection. The evaluation and quantification of the main sampling factors affecting breath analysis output and the development of a breath sampling standard procedure are the most ambitious challenges for the scientific community in this field.

Therefore, this study aimed to compare three different breath sampling approaches in order to deepen advantages and disadvantages among them and to validate two new automatic devices for breath sampling with respect to the most used method consisting of breath collection in polymeric bags. More specifically, a tailored experimental design was developed in order to study the differences in VOCs’ profiles when whole or alveolar breath is collected and when pulmonary wash out with clean air is done. In a framework of three different test sections, coherent and comparable results were obtained using devices both supplied and not supplied by clean air as well as comparing Mistral with the Tedlar bag. On the other hand, VOCs’ levels measured on samples collected by ReCIVA were significantly lower than those detected for Mistral and Tedlar bags, highlighting that a better and online flow control for the ReCIVA system could be advisable. Finally, the analysis of all data including data obtained by explorative analysis of the unique LC breath sample, showed that a clean air supply might determine a further confounding factor in breath analysis because the lung wash out could be incomplete and unequal for all VOCs. On the contrary, the ambient air sampling and the evaluation of changes in VOCs’ levels in breath samples with respect to AA simultaneously collected, could be more useful for a breath analysis purpose with respect to a clean air supply for lung wash out. 

Finally, in order to deepen performance of the two automatic breath sampling devices, a comparison with automatic or manual systems able to collect the alveolar fraction of breath thanks to the CO_2_ control is needed and it should be the main aim for further studies.

## Figures and Tables

**Table 1:** Operative condition of TD-GC/MS analysis.

Step	Parameters	Value
Tube desorption	Purge time	3 min at 5 mL/min–trap in line
	Desorption time	10 min
	Desorption temperature	300 °C
	Temperature of cold trap	20 °C
	Desorption flow	30 mL/min, no split
Focusing trap desorption	Temperature of cold trap desorption	300 °C
	Split low	5 mL/min
	Transfer Line Temperature	200 °C
GC analysis	Gas carrier	He
	Gas flow	1.7 mL/min
	Analytical column	VOCOL^®^ (Supelco), diphenyl dimethyl polysiloxane with crosslinking moieties, 60 m × 0.25 mm ID, 1.5 μm stationary phase thickness
	Oven temperature	37 °C hold for 5 min 37 °C–190 °C at 6 °C/min 190 °C–200 °C at 2 °C/min 200 °C–220 °C at 15 °C/min 220 °C hold for 3 min

## Supplementary Materials

The following are available: Table S1: Mean, Median, and Standard Deviation (STD.DEV) of peak area of the 103 VOCs detected in breath samples collected using Tedlar Bags, Mistral, ReCIVA Low, and ReCIVA Whole from 10 healthy subjects (HC). Number (N) of breath samples in which VOC were detected is also reported. Table S2: Wilcoxon p-values of peak area of the 103 VOCs detected in breath and Ambient Air samples simultaneously collected using Tedlar Bags, Mistral, ReCIVA Low, and ReCIVA Whole. (AA) superscript: higher ambient air levels. (EXP) superscript: higher HC breath sample levels. Table S3: Mean, Median, and Standard Deviation (STD.DEV) of peak area of the 103 VOCs detected in ambient air (AA) samples collected using Mistral and ReCIVA. Number (N) of samples in which VOC were detected is also reported. Alveolar Gradient were calculated as: (Peak Area in Breath sample - Peak Area in AA)/ Peak Area in Breath sample. AA Mistral/AA ReCIVA represents the ratio value between peak area of VOCs detected in ambient air samples simultaneously collected by Mistral and ReCIVA. Table S4: Mean, Median, and Standard Deviation (STD.DEV) of peak area of the 103 VOCs detected in breath samples collected using Tedlar Bags and Mistral. Number (N) of breath samples in which VOC were detected is also reported in addition to the ratio between peak area of VOC detected in samples collected from 10 healthy subjects using Mistral and Bag and to a p-value of the comparison. Table S5: Mean, Median, and Standard Deviation (STD.DEV) of the peak area of the 103 VOCs detected in breath samples collected using RecCIVA Low and ReCIVA Whole. Number (N) of breath samples in which VOC were detected is also reported in addition to the ratio between peak area of VOC detected in samples collected from 10 healthy subjects by using RecCIVA Low and ReCIVA Whole and to p-value of the comparison. Table S6: Peak area of the 103 VOCs detected in breath samples collected using Tedlar Bags, Mistral, ReCIVA Low, and ReCIVA Whole from 1 subject affected by lung cancer (LC) and in ambient air samples collected using Mistral and ReCIVA.
