# Supplementary material for: Breath Analysis: Comparison among Methodological Approaches for Breath Sampling
Source: Molecules. 2020 Dec 10;25(24):5823. doi: 10.3390/molecules25245823 (PMC7763204; doi:10.3390/molecules25245823)
Supplement: Supplementary file 1 [file molecules-25-05823-s001.pdf]

Table S1: Mean, Median and Standard Deviation (STD.DEV) of peak area of the 103 VOCs detected in breath samples collected using Tedlar Bags, Mistral, ReCIVA Low and ReCIVA Whole from 10 healthy subjects (HC). Number (N) of breath samples in which VOC were detected is also reported.

Compound in HC Breath Sample	Tedlar Bags			N	Mistral			N	ReCIVA Low			N	ReCIVA Whole			N
	Mean	STD.DEV	Median		Mean	STD.DEV	Median		Mean	STD.DEV	Median		Mean	STD.DEV	Median	
**1,3-cyclopentadiene**	2.58E+04	3.06E+04	1,33E+04	10	2.26E+04	1.81E+04	2,47E+04	9	1.30E+04	1.42E+04	9,44E+03	7	2.19E+04	2.31E+04	1,41E+04	9
**1-hexanol, 2-ethyl-**	1.06E+06	8.95E+05	7,01E+05	10	2.14E+06	1.27E+06	2,15E+06	10	1.00E+05	4.08E+04	9,04E+04	10	1.34E+05	1.02E+05	8,76E+04	10
**1-octene**	3.13E+05	3.33E+05	2,10E+05	10	2.77E+05	1.81E+05	2,96E+05	9	3.65E+04	5.52E+04	2,24E+04	6	1.90E+05	2.38E+05	9,07E+04	10
**1-propanol**	8.41E+05	6.80E+05	1,04E+06	9	5.88E+05	4.80E+05	5,36E+05	9	1.21E+04	3.81E+04	/	1	7.53E+04	5.42E+04	8,76E+04	8
**1-propanol, 2-butoxy-**	7.84E+03	1.92E+04	/	2	2.05E+04	1.81E+04	2,26E+04	7	/	/	/	0	/	/	/	0
**1-propene, 2methyl-**	1.02E+06	1.06E+06	8,57E+05	8	6.56E+05	8.43E+05	3,83E+05	7	1.24E+05	1.47E+05	6,72E+04	7	3.33E+05	4.45E+05	1,46E+05	6
**2,3-butandione**	4.76E+05	7.28E+05	2,53E+05	10	2.40E+05	1.38E+05	2,08E+05	10	9.09E+04	8.10E+04	8,47E+04	8	3.04E+05	2.54E+05	1,66E+05	10
**2,4-dimethyl heptane**	1.13E+05	1.37E+05	6,03E+04	9	6.22E+03	1.34E+04	/	2	1.67E+03	5.29E+03	/	1	1.87E+04	2.15E+04	9,68E+03	7
**2-butanone**	7.82E+05	1.57E+06	2,91E+05	10	4.99E+05	2.86E+05	4,00E+05	10	8.72E+04	6.09E+04	7,95E+04	9	4.43E+05	3.58E+05	3,40E+05	10
**2-butanone, 3-hydroxy-**	9.04E+04	2.26E+05	8,87E+03	5	8.08E+04	1.19E+05	/	4	2.22E+05	2.08E+05	2,22E+05	7	4.81E+05	1.88E+05	4,16E+05	10
**2-methyl butane - ion 43**	4.58E+05	6.58E+05	3,19E+05	8	2.89E+05	2.87E+05	2,34E+05	7	5.40E+04	9.64E+04	0,00E+00	4	1.11E+05	1.30E+05	4,92E+04	7
**2-methyl butane - ion 57**	3.46E+05	3.18E+05	3,51E+05	8	2.60E+05	1.94E+05	2,38E+05	8	4.24E+04	6.57E+04	1,86E+04	5	1.04E+05	1.43E+05	4,19E+04	7
**2-methyl hexane**	8.72E+04	1.68E+05	3,95E+04	9	3.33E+04	3.23E+04	3,72E+04	6	0.00E+00	0.00E+00	/	0	4.66E+04	7.54E+04	5,96E+03	5
**2-pentanone**	5.46E+05	7.53E+05	2,82E+05	10	3.59E+05	4.95E+05	3,06E+05	8	2.74E+04	6.15E+04	/	3	2.63E+05	2.66E+05	1,99E+05	8
**3-carene**	1.43E+04	2.20E+04	/	4	3.97E+04	1.15E+05	/	3	4.56E+03	7.78E+03	/	3	9.47E+02	2.99E+03	/	1
**3-hexene**	7.43E+03	1.50E+04	/	3	2.56E+03	5.52E+03	3,17E+02	5	/	/	/	0	8.88E+03	1.49E+04	5,08E+02	6
**3-methyl hexane**	4.09E+04	9.02E+04	1,35E+04	7	4.37E+03	7.26E+03	/	3	/	/	/	0	2.20E+04	5.01E+04	/	3
**4-carene**	3.97E+04	3.80E+04	2,12E+04	10	1.94E+04	8.83E+03	1,88E+04	10	1.85E+04	2.07E+04	9,57E+03	10	1.36E+04	1.47E+04	9,62E+03	9

**4-methyl octane**	1.75E+05	8.80E+04	1,41E+05	10	5.40E+04	1.22E+05	/	3	2.15E+04	1.85E+04	2,29E+04	7	4.03E+04	3.73E+04	2,45E+04	10
**acetaldehyde ion44**	3.18E+06	3.70E+06	1,78E+06	9	2.35E+06	2.06E+06	1,49E+06	10	1.37E+06	1.72E+06	1,26E+06	7	5.74E+05	4.75E+05	4,63E+05	9
**Acetaldehyde ion43**	1.86E+06	2.10E+06	1,00E+06	9	1.45E+06	1.19E+06	1,01E+06	10	7.46E+05	9.32E+05	6,57E+05	7	4.22E+05	3.43E+05	3,26E+05	9
**acetone**	9.25E+06	4.04E+06	7,67E+06	10	9.56E+06	5.88E+06	8,09E+06	10	3.40E+06	6.82E+05	3,61E+06	10	2.43E+06	1.26E+06	2,32E+06	10
**acetophenone**	6.38E+06	5.42E+06	5,79E+06	10	8.43E+06	6.94E+06	7,78E+06	10	5.12E+06	5.33E+06	2,97E+06	10	8.63E+06	8.81E+06	5,48E+06	10
**acid acetic methylester – ion 43**	3.64E+05	2.93E+05	2,87E+05	10	2.43E+05	1.20E+05	2,59E+05	9	4.19E+04	4.65E+04	3,12E+04	6	1.06E+05	8.65E+04	8,50E+04	10
**acid acetic methylester – ion 74**	1.13E+05	6.98E+04	1,08E+05	9	8.34E+04	4.30E+04	8,33E+04	9	1.56E+04	1.72E+04	1,10E+04	6	4.18E+04	2.74E+04	3,97E+04	10
**alpha-pinene**	1.39E+05	3.96E+04	1,43E+05	10	1.33E+05	5.90E+04	1,45E+05	10	3.92E+04	2.23E+04	4,18E+04	10	4.65E+04	3.07E+04	3,71E+04	10
**benzaldehyde**	3.83E+06	3.05E+06	3,24E+06	10	5.07E+06	3.17E+06	4,81E+06	10	2.68E+06	2.09E+06	1,94E+06	10	4.03E+06	3.46E+06	2,99E+06	10
**benzene - ion 78**	8.54E+06	1.04E+07	3,78E+06	10	6.62E+06	7.87E+06	2,76E+06	10	1.78E+06	1.60E+06	1,26E+06	10	1.75E+07	1.49E+07	1,19E+07	10
**benzene - ion 52**	1.21E+06	1.46E+06	5,74E+05	10	9.50E+05	1.09E+06	4,06E+05	10	2.65E+05	2.40E+05	1,89E+05	10	2.84E+06	2.77E+06	1,74E+06	10
**benzene, tetramethyl-**	8.83E+04	2.75E+04	8,44E+04	10	9.54E+04	3.50E+04	9,49E+04	10	1.39E+04	7.02E+03	1,08E+04	10	2.25E+04	1.65E+04	1,76E+04	10
**benzoic acid**	3.18E+07	2.64E+07	2,73E+07	10	3.87E+07	2.96E+07	3,72E+07	10	2.65E+07	2.83E+07	1,61E+07	10	3.50E+07	3.44E+07	2,50E+07	10
**benzonitrile**	2.62E+06	2.16E+06	2,92E+06	10	4.51E+06	4.07E+06	3,98E+06	10	2.10E+06	2.12E+06	1,48E+06	10	3.83E+06	4.54E+06	1,94E+06	10
**bromobenzene**	6.67E+04	5.90E+04	7,14E+04	7	9.71E+04	9.33E+04	1,07E+05	6	4.54E+04	5.70E+04	3,08E+04	6	1.14E+05	1.42E+05	6,42E+04	6
**butanal - ion 41**	2.16E+04	3.42E+04	5,22E+03	8	3.77E+03	4.28E+03	2,61E+03	5	1.04E+03	1.74E+03	/	3	2.29E+03	5.12E+03	/	3
**butanal - ion 72**	2.30E+05	4.32E+05	8,70E+04	10	1.60E+05	9.49E+04	1,28E+05	10	2.70E+04	1.87E+04	2,61E+04	9	1.24E+05	9.64E+04	9,13E+04	10
**chlorobenzene**	4.56E+05	6.51E+05	2,94E+05	10	4.86E+05	4.14E+05	5,21E+05	10	1.86E+05	2.11E+05	8,80E+04	10	1.25E+06	2.62E+06	2,94E+05	10
**chloromethane**	1.66E+05	1.15E+05	1,28E+05	10	1.14E+05	9.61E+04	7,26E+04	9	2.24E+04	2.38E+04	1,93E+04	5	1.45E+05	2.35E+05	6,41E+04	10
**ciclopropane, 1,1-dimethyl**	6.80E+04	2.36E+04	6,43E+04	10	4.44E+04	2.33E+04	4,18E+04	10	2.38E+04	1.03E+04	2,14E+04	10	2.44E+04	8.18E+03	2,21E+04	10
**cyclohexane**	2.83E+04	3.76E+04	1,67E+04	10	1.24E+04	6.87E+03	1,33E+04	9	1.94E+03	3.43E+03	/	3	3.35E+04	3.99E+04	1,77E+04	9
**cyclohexane, methyl- - ion 55**	6.97E+04	9.19E+04	4,29E+04	9	4.00E+04	3.26E+04	4,62E+04	7	8.00E+03	1.29E+04	0,00E+00	4	2.67E+04	1.88E+04	2,67E+04	9
**cyclohexane, methyl- - ion 83**	3.94E+04	3.23E+04	3,09E+04	9	2.89E+04	2.48E+04	3,15E+04	7	5.47E+03	1.04E+04	0,00E+00	4	1.35E+04	1.68E+04	4,05E+03	9
**cyclohexanone**	1.46E+05	1.40E+05	1,08E+05	10	2.65E+05	1.67E+05	2,55E+05	10	5.97E+04	3.59E+04	4,94E+04	10	1.01E+05	6.51E+04	9,18E+04	10
**cyclopentane –**	1.49E+06	4.08E+06	/	4	1.01E+05	2.95E+05	/	3	1.60E+05	1.06E+05	1,78E+05	8	1.62E+05	1.02E+05	1,53E+05	9

**ion 42**																
**cyclopentane – ion 70**	5.17E+05	1.60E+06	7,63E+03	7	8.04E+03	9.22E+03	5,24E+03	5	3.31E+04	2.09E+04	3,90E+04	8	3.71E+04	2.29E+04	3,20E+04	9
**cyclopentene, dimethyl-**	5.79E+06	4.42E+06	4,47E+06	9	6.15E+06	5.17E+06	4,89E+06	10	3.52E+06	3.44E+06	2,68E+06	9	4.00E+06	4.47E+06	2,03E+06	10
**decanal**	5.51E+06	2.98E+06	5,28E+06	10	2.69E+06	1.30E+06	2,48E+06	10	4.26E+05	2.43E+05	2,98E+05	10	1.17E+06	2.17E+06	5,09E+05	10
**decane**	1.68E+05	1.09E+05	1,44E+05	10	1.96E+05	9.85E+04	2,30E+05	10	1.04E+05	4.94E+04	1,21E+05	10	1.55E+05	1.19E+05	1,35E+05	10
**decane, 3,7-dimethyl-**	6.10E+05	1.91E+05	6,16E+05	10	1.59E+05	8.20E+04	1,74E+05	10	7.78E+04	3.49E+04	7,13E+04	10	1.31E+05	1.01E+05	9,11E+04	10
**decane, 3,6-dimethyl-**	2.13E+05	8.18E+04	3,13E+05	10	1.33E+04	2.25E+04	/	3	1.50E+04	8.05E+03	7,49E+04	9	2.55E+04	2.15E+04	7,66E+04	9
**dimethyl disulphide**	1.77E+05	2.69E+05	4,61E+04	10	1.89E+05	3.14E+05	6,78E+04	9	1.91E+04	1.65E+04	1,13E+04	10	4.05E+04	6.15E+04	1,69E+04	10
**dimethyl sulphide**	4.27E+05	4.54E+05	2,64E+05	10	3.78E+05	2.90E+05	2,78E+05	10	1.51E+05	1.83E+05	9,14E+04	8	1.20E+05	1.22E+05	7,23E+04	10
**ethanol - ion 45**	8.95E+06	7.75E+06	7,96E+06	10	7.16E+06	4.25E+06	6,57E+06	10	9.87E+05	9.80E+05	6,03E+05	9	6.50E+05	4.78E+05	5,23E+05	10
**ethanol - ion 46**	3.41E+06	3.19E+06	2,86E+06	10	2.51E+06	1.48E+06	2,25E+06	10	3.62E+05	3.49E+05	2,28E+05	9	2.31E+05	1.70E+05	1,80E+05	10
**ethanol - ion 43**	1.88E+06	1.55E+06	1,52E+06	10	1.37E+06	9.10E+05	1,14E+06	10	3.38E+05	3.47E+05	2,62E+05	9	1.04E+05	1.09E+05	6,36E+04	10
**ethyl acetate**	1.95E+05	3.03E+05	8,86E+04	10	7.13E+04	4.32E+04	8,05E+04	9	2.80E+05	3.15E+05	9,54E+04	9	7.62E+05	8.72E+05	4,54E+05	9
**ethylbenzene**	2.02E+05	1.13E+05	1,96E+05	10	5.49E+05	2.22E+05	5,41E+05	10	4.81E+04	2.41E+04	5,12E+04	10	1.00E+05	8.65E+04	7,59E+04	10
**heptanal**	4.85E+05	8.13E+05	2,41E+05	10	3.17E+05	1.64E+05	2,96E+05	10	4.49E+04	2.40E+04	3,47E+04	10	1.24E+05	1.56E+05	7,14E+04	10
**heptane**	7.78E+04	1.58E+05	3,62E+04	8	2.68E+04	2.89E+04	2,14E+04	6	6.10E+03	8.71E+03	2,39E+03	6	2.25E+05	4.17E+05	3,26E+04	8
**hexanal - ion 44**	4.49E+05	7.86E+05	1,97E+05	10	5.60E+05	2.74E+05	4,76E+05	10	4.61E+04	2.05E+04	4,15E+04	10	1.35E+05	1.79E+05	8,44E+04	10
**hexanal - ion 56**	4.51E+05	7.69E+05	2,10E+05	10	6.85E+05	3.24E+05	5,91E+05	10	4.13E+04	2.43E+04	3,38E+04	10	1.38E+05	1.91E+05	7,41E+04	10
**hexane - ion 57**	8.18E+05	7.74E+05	4,79E+05	10	2.22E+05	1.63E+05	2,03E+05	9	3.19E+04	4.12E+04	1,53E+04	6	3.39E+05	7.00E+05	5,44E+04	9
**hexane - ion 86**	1.38E+05	1.33E+05	7,78E+04	10	3.79E+04	2.43E+04	3,35E+04	10	7.88E+03	8.30E+03	5,24E+03	7	6.60E+04	1.37E+05	1,03E+04	8
**ion 46 - rt 15.464**	0.00E+00	0.00E+00	/	0	1.20E+02	3.79E+02	/	1	5.62E+01	1.78E+02	/	1	4.75E+04	1.50E+05	/	1
**ion 56 - rt 9.401**	3.69E+05	4.07E+05	2,37E+05	10	3.37E+05	1.95E+05	3,01E+05	10	1.07E+05	7.68E+04	9,68E+04	9	3.19E+05	3.08E+05	2,05E+05	10
**ion 57 - rt 26.465**	9.58E+03	1.08E+04	6,01E+03	5	1.05E+05	6.52E+04	1,38E+05	10	9.50E+04	4.69E+04	9,27E+04	10	1.08E+05	6.51E+04	1,07E+05	9
**ion 57 - rt 26.488**	6.54E+03	8.91E+03	/	4	1.03E+05	6.60E+04	1,26E+05	9	9.37E+04	4.90E+04	9,15E+04	10	1.20E+05	5.43E+04	1,13E+05	10
**ion 57 - rt 25.056**	2.64E+05	7.59E+04	2,55E+05	10	5.71E+05	3.19E+05	6,74E+05	10	6.17E+05	3.42E+05	6,12E+05	10	7.92E+05	3.80E+05	8,04E+05	10
**ion 57 - rt 36.442**	1.99E+05	9.55E+04	1,90E+05	10	8.70E+05	5.77E+05	1,11E+06	9	3.42E+04	4.39E+04	9,51E+03	5	3.45E+04	5.09E+04	0,00E+00	4
**ion 79 - rt 10.315**	2.07E+07	1.84E+07	1,39E+07	10	1.91E+07	1.43E+07	1,47E+07	10	1.11E+07	7.22E+06	1,04E+07	10	1.39E+07	1.54E+07	8,15E+06	10
**isobutane**	1.72E+05	3.22E+05	/	3	1.14E+05	1.50E+05	/	4	7.40E+03	2.34E+04	/	1	7.00E+03	2.21E+04	/	1
**isoprene**	1.28E+07	5.97E+06	1,37E+07	10	1.49E+07	3.72E+06	1,48E+07	10	3.98E+06	1.67E+06	3,47E+06	10	3.04E+06	6.51E+05	3,07E+06	10

**isopropanol**	2.88E+06	1.58E+06	2,86E+06	10	2.70E+06	1.88E+06	2,07E+06	10	3.02E+05	2.22E+05	3,55E+05	9	3.17E+05	1.80E+05	3,24E+05	10
**isopropyl toluene**	1.01E+05	6.58E+04	7,17E+04	10	1.04E+05	5.56E+04	1,12E+05	10	3.67E+04	2.57E+04	2,45E+04	10	5.03E+04	4.61E+04	2,53E+04	10
**m/p-xilene**	5.85E+05	4.23E+05	5,24E+05	10	1.06E+06	4.11E+05	1,07E+06	10	1.03E+05	6.13E+04	7,13E+04	10	2.06E+05	2.10E+05	1,27E+05	10
**methyl ciclopentane**	5.59E+04	6.75E+04	3,01E+04	10	3.50E+04	2.61E+04	2,87E+04	9	3.49E+03	6.05E+03	/	3	3.26E+04	4.18E+04	2,56E+04	7
**methyl vinyl ketone**	4.53E+05	6.44E+05	2,42E+05	10	4.06E+05	2.59E+05	3,56E+05	10	7.11E+04	4.46E+04	5,84E+04	10	6.05E+05	6.04E+05	4,01E+05	10
**methylene chloride**	4.79E+06	1.48E+07	4,77E+04	9	5.81E+04	5.08E+04	3,74E+04	10	9.52E+04	7.42E+04	1,16E+05	7	1.92E+05	1.98E+05	9,74E+04	10
**naphtalene**	2.70E+05	1.48E+05	2,28E+05	10	3.86E+05	1.84E+05	3,50E+05	10	1.17E+05	7.68E+04	1,00E+05	10	2.04E+05	1.75E+05	1,31E+05	10
**nonanal**	4.11E+06	2.80E+06	3,56E+06	10	3.32E+06	1.68E+06	3,35E+06	10	2.75E+05	1.32E+05	2,16E+05	10	8.69E+05	1.61E+06	3,73E+05	10
**nonane**	3.94E+05	3.55E+05	3,02E+05	10	2.45E+05	1.77E+05	2,17E+05	10	5.58E+04	4.64E+04	3,63E+04	10	2.19E+05	3.08E+05	8,16E+04	10
**octanal - ion 43**	1.44E+06	1.44E+06	1,03E+06	10	6.82E+05	3.46E+05	6,87E+05	10	1.33E+05	6.74E+04	1,04E+05	10	3.62E+05	5.21E+05	2,08E+05	10
**octanal - ion 57**	1.21E+06	1.16E+06	8,87E+05	10	5.93E+05	3.01E+05	6,11E+05	10	1.29E+05	8.27E+04	9,22E+04	10	3.24E+05	4.47E+05	1,99E+05	10
**octane**	3.11E+05	3.28E+05	2,04E+05	10	2.46E+05	1.80E+05	2,20E+05	9	3.49E+04	5.54E+04	1,41E+04	5	1.89E+05	2.34E+05	9,14E+04	10
**o-xilene**	1.59E+05	9.70E+04	1,37E+05	10	4.08E+05	1.81E+05	4,11E+05	10	2.21E+04	1.17E+04	1,98E+04	10	5.48E+04	6.37E+04	3,07E+04	10
**para-benzoquinone**	1.46E+05	1.35E+05	1,62E+05	7	9.99E+04	8.26E+04	1,16E+05	7	2.78E+04	4.91E+04	1,12E+04	7	3.64E+05	4.71E+05	1,47E+05	8
**pentanal**	4.04E+05	1.00E+06	9,74E+04	9	2.13E+05	2.02E+05	1,79E+05	7	1.28E+04	2.07E+04	/	3	1.08E+05	9.52E+04	7,55E+04	10
**pentane - ion 43**	2.70E+05	1.91E+05	2,30E+05	9	3.14E+05	2.77E+05	2,47E+05	8	4.29E+04	6.86E+04	1,55E+04	5	9.76E+04	1.21E+05	5,58E+04	9
**pentane - ion 57**	6.99E+04	4.51E+04	7,52E+04	9	6.78E+04	5.41E+04	5,85E+04	8	9.97E+03	1.61E+04	3,82E+03	5	2.52E+04	2.98E+04	1,46E+04	9
**pentane, 2-methyl- - ion 43**	2.19E+05	1.33E+05	2,57E+05	9	1.90E+05	1.58E+05	1,77E+05	8	2.21E+04	3.58E+04	0,00E+00	4	6.24E+04	1.01E+05	1,74E+04	6
**pentane, 2-methyl- - ion 71**	1.44E+05	8.53E+04	1,52E+05	9	1.32E+05	6.31E+04	1,35E+05	10	1.20E+04	1.60E+04	5,93E+03	5	4.32E+04	5.76E+04	2,04E+04	7
**phenylethyne**	1.74E+06	1.51E+06	1,79E+06	10	2.32E+06	1.80E+06	2,24E+06	10	7.97E+05	6.72E+05	5,15E+05	10	2.93E+06	3.00E+06	2,44E+06	10
**ion 57 - rt 14.989**	5.54E+03	9.82E+03	/	4	2.35E+03	3.88E+03	/	3	1.49E+03	4.72E+03	/	1	1.89E+04	2.62E+04	5,06E+03	5
**propanoic acid**	5.22E+05	1.31E+06	2,30E+04	6	7.57E+04	9.97E+04	1,97E+04	5	0.00E+00	0.00E+00	/	0	1.81E+05	2.48E+05	0,00E+00	4
**propylbenzene**	7.22E+04	4.72E+04	5,67E+04	10	1.25E+05	6.51E+04	1,36E+05	10	1.79E+04	1.27E+04	1,47E+04	9	3.84E+04	3.75E+04	2,40E+04	10
**sevoflurane**	8.85E+04	1.11E+05	7,14E+04	8	1.78E+05	1.31E+05	1,14E+05	10	1.49E+04	1.68E+04	1,12E+04	6	1.75E+04	1.65E+04	1,36E+04	7
**styrene**	2.13E+05	1.44E+05	1,78E+05	10	5.13E+05	2.87E+05	4,43E+05	10	1.25E+05	1.01E+05	7,96E+04	10	2.32E+05	2.01E+05	1,56E+05	10
**tetrachloroethylene**	1.25E+05	5.35E+04	1,10E+05	10	2.08E+05	7.50E+04	1,94E+05	10	3.08E+04	1.75E+04	2,77E+04	10	9.77E+04	1.47E+05	3,62E+04	10
**tetradecane**	2.32E+05	2.33E+05	1,63E+05	10	4.90E+05	2.17E+05	4,50E+05	10	8.81E+04	6.44E+04	6,67E+04	10	1.28E+05	1.10E+05	8,03E+04	10
**toluene**	1.15E+06	7.30E+05	1,04E+06	10	1.14E+06	4.97E+05	1,27E+06	10	1.97E+05	1.35E+05	1,35E+05	10	1.02E+06	1.53E+06	2,69E+05	10
**trichloromethane**	1.34E+05	3.31E+05	3,04E+04	10	6.55E+03	4.42E+03	6,26E+03	9	5.11E+02	1.55E+03	/	2	1.84E+04	4.16E+04	4,42E+03	8

**undecane, 5-methyl**	1.33E+05	4.20E+05	/	1	2.34E+05	5.04E+05	/	2	1.90E+04	4.03E+04	/	2	4.74E+04	1.19E+05	/	2
**ion 43 - rt 7.343**	1.60E+06	1.67E+06	9,82E+05	9	1.12E+06	9.03E+05	7,64E+05	10	6.12E+05	5.69E+05	5,59E+05	7	7.12E+05	6.82E+05	3,97E+05	10
**ion 43 - rt 5.888**	2.27E+06	1.84E+06	1,86E+06	10	1.81E+06	1.90E+06	1,05E+06	10	1.69E+05	1.89E+05	9,98E+04	9	5.44E+05	6.09E+05	3,21E+05	9

Table 2. Wilcoxon p-values of peak area of the 103 VOCs detected in breath and Ambient Air samples simultaneously collected using Tedlar Bags, Mistral, ReCIVA Low and ReCIVA Whole. (AA) superscript: higher ambient air levels; (EXP) superscript: higher HC breath sample levels.

Compounds	p-value AA vs EXP Mistral	p-value AA vs EXP ReCIVA Low	p-value AA vs EXP ReCIVA Whole	p-value AA vs EXP Bag
**1,3-cyclopentadiene**	0,375	0,153	0,492	0,625
**1-hexanol, 2-ethyl-**	***0,049***(AA)	***0,002***(AA)	0,232	***0,002***(AA)
**1-octene**	***0,049***(AA)	***0,014***(AA)	0,770	***0,049***(AA)
**1-propanol**	***0,004***(EXP)	1,000	***0,014***(EXP)	***0,009***(EXP)
**1-propanol, 2-butoxy-**	0,673			0,371
**1-propene, 2-methyl-**	0,294	0,193	0,624	0,097
**2,3-butandione**	0,064	***0,004*** (AA)	0,557	0,193
**2,4-dimethyl heptane**	***0,010***(AA)	***0,008***(AA)	***0,002***(AA)	0,131
**2-butanone**	***0,002***(AA)	***0,002***(AA)	0,084	0,084
**2-butanone, 3-hydroxy-**	***0,049***(AA)	0,770	***0,006***(AA)	0,083
**2-methyl butane - ion 43**	***0,004***(AA)	***0,006***(AA)	***0,008***(AA)	0,322
**2-methyl butane - ion 57**	***0,027***(AA)	***0,006***(AA)	0,221	0,084
**2-methyl hexane**	***0,002***(AA)	0,098	0,281	***0,024***(AA)
**2-pentanone**	0,770	0,052	0,131	***0,006***(EXP)
**3-carene**	0,281	0,181	1,000	0,100
**3-hexene**	***0,013***(AA)	***0,006***(AA)	0,432	0,126
**3-methyl hexane**	***0,002***(AA)	***0,009***(AA)	0,236	0,275
**4-carene**	0,922	***0,002***(EXP)	***0,037***(EXP)	***0,002***(EXP)
**4-methyl octane**	0,800	***0,002*** (AA)	***0,037***(AA)	***0,010***(AA)

**acetaldehyde - ion 44**	***0,004***$^{(AA)}$	***0,037***$^{(AA)}$	***0,002***$^{(AA)}$	**0,015**$^{(AA)}$
**acetaldehyde - ion 43**	***0,002***$^{(AA)}$	***0,027***$^{(AA)}$	***0,002***$^{(AA)}$	**0,016**$^{(AA)}$
**acetone**	***0,002***$^{(EXP)}$	***0,002***$^{(EXP)}$	***0,002***$^{(EXP)}$	***0,002***$^{(EXP)}$
**acetophenone**	**0,027**$^{(EXP)}$	0,375	0,922	0,922
**acid acetic methylester - ion 43**	1,000	***0,013***$^{(AA)}$	0,492	0,432
**acid acetic methylester - ion 74**	0,846	***0,024***$^{(AA)}$	0,557	0,275
**alpha-pinene**	0,193	0,695	0,432	***0,002***
**benzaldehyde**	0,432	***0,049***$^{(AA)}$	0,432	0,770
**benzene - ion 78**	***0,006***$^{(AA)}$	***0,002***$^{(AA)}$	**0,022**$^{(AA)}$	**0,048**$^{(AA)}$
**benzene - ion 52**	***0,006***$^{(AA)}$	***0,002***$^{(AA)}$	**0,022**$^{(AA)}$	**0,049**$^{(AA)}$
**benzene, tetramethyl-**	1,000	0,770	0,131	***0,002***$^{(AA)}$
**benzoic acid**	**0,004**$^{(EXP)}$	**0,013**$^{(EXP)}$	**0,043**$^{(AA)}$	1,000
**benzonitrile**	***0,004***$^{(EXP)}$	0,922	***0,037***$^{(AA)}$	0,492
**bromobenzene**	1,000	0,126	0,635	0,922
**butanal - ion 41**	***0,002***$^{(AA)}$	0,281	0,787	0,141
**butanal - ion 72**	***0,002***$^{(AA)}$	***0,002***$^{(AA)}$	***0,049***$^{(AA)}$	0,084
**chlorobenzene**	***0,014***$^{(AA)}$	***0,037***$^{(AA)}$	0,492	0,846
**chloromethane**	0,695	***0,044***$^{(AA)}$	0,922	***0,004***$^{(AA)}$
**ciclopropane, 1,1-dimethyl-2-(3methyl-1)**	0,770	***0,002***$^{(EXP)}$	***0,002***$^{(EXP)}$	***0,002***$^{(EXP)}$
**cyclohexane**	***0,002***$^{(AA)}$	***0,006***$^{(AA)}$	0,922	0,193
**cyclohexane, methyl- - ion 55**	0,160	***0,008***$^{(AA)}$	0,922	***0,037***$^{(AA)}$
**cyclohexane, methyl- - ion 83**	0,084	***0,025***$^{(AA)}$	0,375	***0,027***$^{(AA)}$
**cyclohexanone**	0,922	0,193	0,322	***0,004***$^{(AA)}$
**cyclopentane - ion 42**	0,272	***0,042***$^{(AA)}$	0,232	0,812
**cyclopentane - ion 70**	0,236	***0,021***$^{(AA)}$	0,160	0,155
**cyclopentene, dimethyl-**	***0,027***$^{(EXP)}$	**0,007**$^{(EXP)}$	0,922	0,160
**decanal**	0,160	***0,020***$^{(AA)}$	0,625	***0,002***$^{(EXP)}$
**decane**	***0,031***$^{(AA)}$	***0,049***$^{(AA)}$	0,275	0,695
**decane, 3,7-dimethyl-**	0,084	0,131	1,000	***0,002***$^{(AA)}$
**decane, dimethyl-**	***0,022***$^{(AA)}$	0,160	0,846	***0,002***$^{(AA)}$
**dimethyl disulphide**	***0,004***$^{(EXP)}$	***0,010***$^{(EXP)}$	***0,014***$^{(EXP)}$	***0,002***$^{(EXP)}$
**dimethyl sulphide**	***0,002***$^{(EXP)}$	***0,013***$^{(EXP)}$	***0,002***$^{(EXP)}$	***0,002***$^{(EXP)}$
**ethanol - ion 45**	***0,014***$^{(AA)}$	***0,002***$^{(AA)}$	***0,002***$^{(AA)}$	0,275
**ethanol - ion 46**	***0,014***$^{(AA)}$	***0,002***$^{(AA)}$	***0,002***$^{(AA)}$	0,275
**ethanol - ion 43**	***0,049***$^{(AA)}$	***0,004***$^{(AA)}$	***0,002***$^{(AA)}$	0,160
**ethyl acetate**	***0,002***$^{(AA)}$	***0,009***$^{(AA)}$	***0,009***$^{(AA)}$	***0,004***$^{(AA)}$
**ethylbenzene**	***0,049***$^{(AA)}$	***0,002***$^{(AA)}$	***0,020***$^{(AA)}$	0,557

**heptanal**	***0,027***(AA)	***0,004***(AA)	0,557	***0,002***(AA)
**heptane**	***0,002***(AA)	***0,002***(AA)	0,922	0,232
**hexanal - ion 44**	***0,020***(AA)	***0,002***(AA)	0,105	0,084
**hexanal - ion 56**	***0,020***(AA)	***0,002***(AA)	0,105	***0,049***(AA)
**hexane - ion 57**	***0,027*** ((AA)	***0,002*** (AA)	0,846	***0,002*** (AA)
**hexane - ion 86**	***0,027*** (AA)	***0,010*** (AA)	1,000	***0,002***(AA)
**ion 46 - rt 15.464**	0,423	0,414	1,000	0,346
**ion 56 - rt 9.401**	0,492	***0,002***(AA)	0,432	0,770
**ion 57 - rt 26.465**	0,084	***0,037***(AA)	***0,049***(AA)	***0,006***(AA)
**ion 57 - rt 26.488**	0,064	***0,037***(AA)	***0,037***(AA)	***0,006***(AA)
**ion 57 - rt 25.056**	0,084	0,064	0,084	***0,002***
**ion 57 - rt 36.442**	0,064	***0,014***(AA)	***0,014***(AA)	0,695
**ion 79 - rt 10.315**	0,625	***0,006***(EXP)	***0,002***(EXP)	***0,002***(EXP)
**isobutane**	0,590	1,000	1,000	0,181
**isoprene**	***0,002***(EXP)	***0,002***(EXP)	***0,002***(EXP)	***0,002***(EXP)
**isopropanol**	0,846	***0,027***(AA)	***0,027***(AA)	***0,010***(AA)
**isopropyl toluene**	0,064	0,695	0,625	***0,004***(AA)
**m/p-xilene**	***0,027***(AA)	***0,002***(AA)	***0,037***(AA)	0,064
**methyl ciclopentane**	***0,002***(AA)	***0,006***(AA)	0,683	0,695
**methyl vinyl ketone**	0,770	***0,006***(AA)	0,193	0,322
**methylene chloride**	0,105	***0,002***(AA)	***0,014***(AA)	***0,004***(AA)
**naphtalene**	0,193	***0,002***(AA)	0,770	0,084
**nonanal**	0,084	***0,002***(AA)	0,084	***0,002***(EXP)
**nonane**	***0,049***(AA)	***0,020***(AA)	0,922	***0,002***(EXP)
**octanal - ion 43**	***0,037***(AA)	***0,006***(AA)	0,432	***0,002***(EXP)
**octanal - ion 57**	0,160	***0,049***(AA)	0,625	***0,002***(EXP)
**octane**	***0,014***(AA)	***0,014***(AA)	0,846	***0,049***(AA)
**o-xilene**	0,160	***0,002***(AA)	***0,049***(AA)	0,322
**para-benzoquinone**	0,076	***0,004***	0,275	0,083
**pentanal**	0,375	***0,006***	0,846	0,557
**pentane - ion 43**	1,000	***0,022***	0,554	0,084
**pentane - ion 57**	0,624	***0,022***	0,813	***0,004***(AA)
**pentane, 2-methyl- - ion 43**	***0,002***(AA)	***0,022***(AA)	0,151	***0,049***(AA)
**pentane, 2-methyl- - ion 71**	***0,020***(AA)	***0,014***(AA)	0,107	***0,027***(AA)
**phenylethyne**	0,432	***0,020***(AA)	0,695	0,375
**ion 57 - rt 14.989**	***0,002***(AA)	***0,006***(AA)	***0,014***(AA)	***0,008***(AA)
**propanoic acid**	***0,002***(AA)	***0,006***(AA)	***0,006***(AA)	***0,006***(AA)

**propylbenzene**	***0,049***(AA)	***0,002***(AA)	0,432	***0,037***(AA)
**sevoflurane**	***0,037***(AA)	***0,006***(AA)	***0,006***(AA)	0,695
**styrene**	***0,006***(AA)	***0,006***(AA)	0,275	0,160
**tetrachloroethylene**	***0,020***(AA)	***0,002***(AA)	0,432	0,193
**tetradecane**	0,232	0,322	0,375	***0,004***(AA)
**toluene**	***0,004***(AA)	***0,002***(AA)	0,432	0,275
**trichloromethane**	***0,002***(AA)	***0,006***(AA)	0,084	0,193
**undecane, 5-methyl-**	0,584	0,423	1,000	1,000
**ion 43 - rt 7.343**	0,375	***0,006***(EXP)	0,375	0,492
**ion 43 - rt 5.888**	0,432	0,695	0,770	0,193

Table 3. Mean, Median and Standard Deviation (STD.DEV) of peak area of the 103 VOCs detected in ambient air (AA) samples collected using Mistral and ReCIVA. Number (N) of samples in which VOC were detected is also reported. Alveolar Gradient were calculated as: (Peak Area in Breath sample - Peak Area in AA)/ Peak Area in Breath sample. AA Mistral/AA ReCIVA represents the ratio value between peak area of VOCs detected in ambient air samples simultaneously collected by Mistral and ReCIVA.

**Compound in Ambient Air Sample**	**Mistral**			**N**	**ReCIVA**			**N**	**Mistral Alveolar Gradient**		**ReCIVA Alveolar Gradient**		**AA Mistral/AA ReCIVA**
	**Mean**	**STD.DEV**	**Median**		**Mean**	**STD.DEV**	**Median**		**Mean**	**STD.DEV**	**Mean**	**STD.DEV**	
**1,3-cyclopentadiene**	2,77E+04	2,60E+04	1,75E+04	10	1,83E+04	1,17E+04	1,87E+04	10	-0,3	0,6	-1,2	2,1	2
**1-hexanol, 2-ethyl-**	2,71E+06	1,97E+06	2,51E+06	10	1,54E+05	7,45E+04	1,33E+05	10	-0,2	0,5	-0,6	0,5	18
**1-octene**	5,21E+05	4,76E+05	3,94E+05	10	1,74E+05	1,28E+05	1,54E+05	10	-0,7	0,9	-2,4	2,4	3
**1-propanol**	6,02E+04	4,16E+04	6,08E+04	10	1,95E+03	4,11E+03	/	2	0,8	0,1	1,0	/	
**1-propanol, 2-butoxy-**	1,89E+04	2,09E+04	1,24E+04	5	/	/	0,00E+00	/	0,03	0,7	/	/	
**1-propene, 2-methyl-**	6,66E+05	7,73E+05	3,64E+05	6	3,32E+05	3,51E+05	2,22E+05	7	-0,3	1,1	-2,6	3,6	2
**2,3-butandione**	3,91E+05	2,77E+05	2,80E+05	10	2,08E+05	7,34E+04	1,74E+05	10	-0,9	0,8	-1,1	1,1	2
**2,4-dimethyl heptane**	4,09E+04	2,81E+04	2,82E+04	10	4,87E+04	3,71E+04	4,32E+04	10	0,1	0,1	0,0	/	1
**2-butanone**	2,06E+06	1,52E+06	1,55E+06	10	7,33E+05	4,20E+05	6,13E+05	10	-3,2	1,8	-8,2	9,3	3
**2-butanone, 3-hydroxy-**	1,66E+05	1,19E+05	1,20E+05	10	2,45E+05	6,88E+04	2,43E+05	10	0,1	0,1	-0,03	0,9	1
**2-methyl butane - ion 43**	9,78E+05	8,74E+05	8,15E+05	10	4,95E+05	3,82E+05	5,46E+05	10	-2,6	2,5	-1,1	1,2	2

**2-methyl butane - ion 57**	4,05E+05	2,48E+05	3,80E+05	10	1,53E+05	4,67E+04	1,41E+05	10	-0,6	0,8	-2,0	1,5	3
**2-methyl hexane**	1,97E+05	9,52E+04	2,20E+05	10	1,70E+04	2,29E+04	/	4	-4,0	1,2	/	/	12
**2-pentanone**	3,25E+05	2,57E+05	2,30E+05	10	1,22E+05	3,81E+04	1,09E+05	10	-0,6	1,3	-2,9	4,3	3
**3-carene**	2,09E+03	4,41E+03	/	2	/	/	0,00E+00	/	/	/	/	/	/
**3-hexene**	1,67E+04	1,76E+04	8,55E+03	9	1,09E+04	1,03E+04	9,75E+03	10	-8,5	8,0	/	/	2
**3-methyl hexane**	1,02E+05	4,76E+04	1,15E+05	10	2,77E+04	1,69E+04	2,75E+04	9	-9,2	1,9	/	/	4
**4-carene**	2,01E+04	1,19E+04	1,47E+04	10	1,21E+03	3,83E+03	/	1	-0,1	0,6	0,9	0,2	/
**4-methyl octane**	3,91E+04	4,11E+04	2,70E+04	6	6,70E+04	4,18E+04	4,93E+04	10	0,7	0,3	-1,5	1,7	1
**acetaldehyde ion 44**	6,43E+06	3,97E+06	4,83E+06	10	3,46E+06	2,10E+06	3,23E+06	10	-2,2	2,1	-1,4	1,9	2
**acetaldehyde ion 43**	3,59E+06	2,31E+06	2,67E+06	10	1,89E+06	1,16E+06	1,71E+06	10	-2,8	2,4	-1,6	2,2	2
**acetone**	1,52E+06	7,82E+05	1,20E+06	10	3,56E+05	8,91E+04	3,10E+05	10	0,8	0,1	0,9	0,0	4
**acetophenone**	7,49E+06	7,85E+06	3,74E+06	10	6,11E+06	5,19E+06	5,20E+06	10	0,2	0,4	-0,8	1,8	1
**acid acetic methylester – ion 43**	3,03E+05	2,91E+05	1,96E+05	10	2,13E+05	2,10E+05	1,18E+05	9	-0,2	0,8	-2,6	4,3	1
**acid acetic methylester – ion 74**	8,95E+04	7,32E+04	5,80E+04	10	6,66E+04	5,38E+04	4,47E+04	9	0,01	0,6	-2,1	2,9	1
**alpha-pinene**	1,50E+05	7,58E+04	1,29E+05	10	3,39E+04	1,34E+04	3,20E+04	10	-0,2	0,5	-0,3	1,1	4
**benzaldehyde**	5,92E+06	5,34E+06	4,24E+06	10	4,09E+06	2,90E+06	3,96E+06	10	-0,1	0,4	-0,7	0,8	1
**benzene - ion 78**	1,77E+07	1,40E+07	1,26E+07	10	1,58E+07	1,00E+07	1,73E+07	10	-3,8	2,7	-10,5	7,1	1
**benzene - ion 52**	2,78E+06	2,41E+06	1,84E+06	10	2,31E+06	1,48E+06	2,51E+06	10	-3,7	2,7	-10,7	7,2	1
**benzene, tetramethyl-**	9,77E+04	6,06E+04	9,05E+04	10	1,42E+04	7,14E+03	1,31E+04	9	0,04	0,4	-0,2	0,6	7
**benzoic acid**	3,77E+07	3,57E+07	2,31E+07	10	3,38E+07	3,18E+07	2,97E+07	10	0,1	0,4	-0,8	1,4	1
**benzonitrile**	2,92E+06	3,15E+06	1,53E+06	10	2,11E+06	2,14E+06	1,90E+06	10	0,4	0,3	-0,1	1,0	1
**bromobenzene**	9,88E+04	1,14E+05	8,19E+04	6	8,35E+04	9,43E+04	7,56E+04	8	0,1	0,5	-1,1	2,9	1
**butanal - ion 41**	4,22E+04	3,17E+04	3,24E+04	10	3,09E+03	5,02E+03	/	3	-4,1	2,8	-0,5	2,6	/
**butanal - ion 72**	5,81E+05	4,38E+05	4,46E+05	10	2,03E+05	1,13E+05	1,73E+05	10	-2,6	1,5	-7,1	7,9	3
**chlorobenzene**	3,79E+05	3,75E+05	3,00E+05	10	4,71E+05	5,45E+05	3,45E+05	10	0,05	0,6	-5,7	11,9	1
**chloromethane**	8,44E+04	7,20E+04	5,50E+04	9	6,40E+04	3,92E+04	5,59E+04	9	-0,03	1,0	-0,6	1,0	1
**ciclopropane, 1,1-dimethyl**	3,95E+04	2,28E+04	2,98E+04	10	4,31E+03	5,37E+03	4,20E+03	6	0,03	0,4	0,8	0,2	9
**cyclohexane**	8,80E+04	5,48E+04	6,23E+04	10	2,95E+04	1,22E+04	2,51E+04	10	-6,4	6,1	-3,9	3,1	3
**cyclohexane,**	5,06E+04	2,15E+04	4,53E+04	10	2,97E+04	1,99E+04	1,90E+04	10	-0,1	0,4	-0,6	0,7	2

**methyl- ion 55**													
**cyclohexane, methyl- ion 83**	4,17E+04	1,99E+04	4,44E+04	10	1,76E+04	5,31E+03	1,82E+04	10	-0,3	0,7	-0,5	0,9	2
**cyclohexanone**	2,76E+05	1,91E+05	2,46E+05	10	7,76E+04	2,69E+04	6,84E+04	10	-0,04	0,3	-0,7	1,1	4
**cyclopentane – ion 42**	5,58E+04	5,96E+04	4,50E+04	7	2,78E+05	2,54E+05	2,25E+05	8	0,1	0,7	-0,8	1,0	0,2
**cyclopentane – ion 70**	1,70E+04	1,77E+04	1,98E+04	7	5,85E+04	4,95E+04	5,10E+04	8	0,1	0,9	-0,8	0,7	0,3
**cyclopentene, dimethyl-**	3,14E+06	3,26E+06	1,47E+06	10	2,92E+06	2,04E+06	2,71E+06	10	0,2	0,9	0,1	1,2	1
**decanal**	3,00E+06	1,77E+06	2,67E+06	10	7,05E+05	3,52E+05	5,37E+05	10	-0,1	0,3	-1,0	1,4	4
**decane**	2,55E+05	1,51E+05	2,25E+05	10	1,74E+05	8,45E+04	1,58E+05	10	-0,4	0,6	-1,0	1,1	1
**decane, 3,7-dimethyl-**	2,05E+05	1,40E+05	1,80E+05	10	1,18E+05	5,45E+04	1,07E+05	10	-0,3	0,4	-0,9	1,2	2
**decane, 3,6-dimethyl-**	3,63E+04	4,22E+04	0,00E+00	7	2,36E+04	1,14E+04	8,77E+04	10	-1,2	0,7	-0,9	1,9	2
**dimethyl disulphide**	6,80E+03	8,11E+03	6,21E+03	7	2,12E+03	3,44E+03	/	3	0,9	0,1	0,7	0,7	3
**dimethyl sulphide**	5,31E+04	3,68E+04	3,98E+04	10	8,58E+03	7,11E+03	9,15E+03	7	0,8	0,2	0,9	0,1	6
**ethanol - ion 45**	1,76E+07	1,34E+07	1,19E+07	10	5,16E+06	2,56E+06	4,37E+06	10	-2,0	2,7	-4,2	7,4	3
**ethanol - ion 46**	6,51E+06	4,87E+06	4,51E+06	10	1,94E+06	9,51E+05	1,69E+06	10	-1,9	2,2	-7,9	9,3	3
**ethanol - ion 43**	3,16E+06	2,36E+06	2,04E+06	10	8,59E+05	5,88E+05	8,12E+05	10	-2,0	2,1	-7,1	7,5	4
**ethyl acetate**	4,33E+05	2,25E+05	3,61E+05	10	3,49E+06	1,86E+06	4,18E+06	9	-5,2	2,5	-33	37	0,1
**ethylbenzene**	8,08E+05	3,92E+05	7,04E+05	10	2,28E+05	1,03E+05	1,89E+05	10	-0,5	0,4	-5,5	4,6	4
**heptanal**	4,45E+05	2,93E+05	3,25E+05	10	1,02E+05	3,86E+04	8,67E+04	10	-0,4	0,4	-2,1	2,3	4
**heptane**	2,26E+05	1,95E+05	1,62E+05	10	9,99E+04	9,31E+04	8,30E+04	10	-3,5	2,6	-27	49	2
**hexanal - ion 44**	8,83E+05	5,03E+05	6,39E+05	10	1,59E+05	6,03E+04	1,42E+05	10	-0,6	0,5	-3,0	2,1	6
**hexanal - ion 56**	1,11E+06	6,25E+05	7,85E+05	10	1,61E+05	5,69E+04	1,45E+05	10	-0,7	0,5	-4,3	3,8	7
**hexane - ion 57**	4,53E+05	4,00E+05	4,20E+05	10	1,60E+05	1,70E+05	1,10E+05	10	-0,9	1,3	-3,9	2,6	3
**hexane - ion 86**	7,88E+04	6,94E+04	7,50E+04	10	2,84E+04	3,03E+04	2,03E+04	10	-1,0	1,5	-2,4	2,2	3
**ion 46 - rt 15.464**	3,37E+03	7,86E+03	/	2	8,59E+02	1,81E+03	/	2	1,0	NA	1,0	NA	4
**ion 56 - rt 9.401**	4,11E+05	3,00E+05	2,81E+05	10	2,61E+05	7,61E+04	2,47E+05	10	-0,4	0,8	-2,1	3,0	2
**ion 57 - rt 26.465**	1,30E+05	8,83E+04	1,12E+05	10	1,66E+05	6,73E+04	1,47E+05	10	-0,4	0,5	-1,3	1,5	1
**ion 57 - rt 26.488**	1,26E+05	8,60E+04	1,15E+05	10	1,64E+05	6,83E+04	1,45E+05	10	-0,2	0,4	-1,4	1,6	1
**ion 57 - rt 25.056**	7,16E+05	4,67E+05	6,16E+05	10	1,01E+06	4,81E+05	8,98E+05	10	-0,3	0,5	-1,2	1,5	1
**ion 57 - rt 36.442**	1,04E+06	7,92E+05	1,15E+06	10	2,59E+05	3,46E+05	1,54E+05	8	-0,2	0,4	-4,9	3,1	4
**ion 79 - rt 10.315**	2,01E+07	1,87E+07	1,20E+07	10	4,84E+06	3,03E+06	3,54E+06	10	-0,02	0,3	0,5	0,3	4
**isobutane**	9,60E+04	2,43E+05	/	2	/	/	/	/					
**isoprene**	5,94E+05	4,45E+05	4,42E+05	10	1,08E+05	8,30E+04	7,83E+04	9	1,0	0,0	1,0	0,02	6

**isopropanol**	2,71E+06	1,91E+06	2,22E+06	10	7,90E+05	5,28E+05	7,21E+05	10	-0,1	0,8	-22	61,9	3
**isopropyl toluene**	1,47E+05	1,01E+05	1,38E+05	10	3,19E+04	1,09E+04	3,40E+04	10	-0,4	0,5	-0,1	0,6	5
**m/p-xilene**	1,57E+06	6,83E+05	1,50E+06	10	3,97E+05	7,46E+04	4,05E+05	10	-0,6	0,4	-3,9	2,4	4
**methyl ciclopentane**	1,17E+05	1,07E+05	8,34E+04	10	3,38E+04	2,53E+04	2,05E+04	10	-2,1	1,5	-2,8	2,0	3
**methyl vinyl ketone**	3,94E+05	2,79E+05	2,52E+05	10	1,95E+05	6,26E+04	1,85E+05	10	-0,2	0,7	-2,4	1,7	2
**methylene chloride**	1,12E+05	8,11E+04	9,28E+04	10	2,72E+05	1,48E+05	2,27E+05	10	-2,0	3,1	-1,0	1,2	0,4
**naphtalene**	4,38E+05	2,86E+05	3,80E+05	10	1,85E+05	7,47E+04	1,59E+05	10	-0,1	0,3	-0,8	0,8	2
**nonanal**	3,95E+06	2,45E+06	3,40E+06	10	7,02E+05	2,83E+05	6,23E+05	10	-0,2	0,3	-2,1	2,2	6
**nonane**	3,26E+05	2,73E+05	2,30E+05	10	1,61E+05	1,29E+05	1,51E+05	10	-0,4	0,5	-3,1	3,0	2
**octanal - ion 43**	9,16E+05	6,62E+05	6,65E+05	10	2,81E+05	1,48E+05	2,38E+05	10	-0,3	0,5	-1,7	2,2	3
**octanal - ion 57**	7,35E+05	5,31E+05	5,35E+05	10	2,43E+05	1,66E+05	2,09E+05	10	-0,2	0,4	-1,7	2,9	3
**octane**	5,44E+05	5,08E+05	3,94E+05	10	1,75E+05	1,29E+05	1,59E+05	10	-0,9	0,8	-2,0	2,4	3
**o-xilene**	5,45E+05	3,27E+05	4,42E+05	10	1,21E+05	1,82E+04	1,28E+05	10	-0,4	0,7	-5,8	3,6	4
**para-benzoquinone**	1,28E+06	1,47E+06	8,61E+05	6	7,83E+05	8,34E+05	5,88E+05	10	-12	10,7	-93	139	2
**pentanal**	2,69E+05	1,67E+05	2,01E+05	10	9,63E+04	5,23E+04	9,44E+04	10	-0,3	1,0	-1,0	0,8	3
**pentane - ion 43**	2,72E+05	2,98E+05	1,93E+05	5	1,40E+05	1,23E+05	1,59E+05	7	-0,1	1,1	-3,5	2,4	2
**pentane - ion 57**	7,54E+04	6,41E+04	7,87E+04	8	2,76E+04	2,15E+04	3,54E+04	7	-0,1	0,6	-2,3	1,5	3
**pentane, 2-methyl- ion 43**	4,72E+05	3,31E+05	4,82E+05	10	1,34E+05	1,28E+05	1,06E+05	7	-1,4	1,2	-4,2	1,7	4
**pentane, 2-methyl- ion 71**	2,29E+05	1,46E+05	2,34E+05	10	8,10E+04	6,35E+04	6,28E+04	8	-0,7	0,8	-4,7	2,6	3
**phenylethyne**	3,15E+06	3,26E+06	2,11E+06	10	2,62E+06	2,60E+06	2,10E+06	10	-0,2	0,7	-2,9	3,0	1
**ion 57 - rt 14.989**	3,46E+04	2,74E+04	3,16E+04	10	6,37E+04	1,12E+05	3,02E+04	10	-6,7	7,1	-25	/	1
**propanoic acid**	4,86E+05	4,33E+05	3,36E+05	10	3,83E+04	1,68E+04	3,28E+04	10	-8,4	13,3	/	/	13
**propylbenzene**	1,68E+05	9,26E+04	1,75E+05	10	4,48E+04	1,56E+04	4,22E+04	10	-0,4	0,5	-1,6	1,0	4
**sevoflurane**	2,98E+05	3,22E+05	1,49E+05	10	1,17E+05	1,17E+05	7,60E+04	10	-0,7	0,9	-7,0	8,9	3
**styrene**	7,60E+05	4,77E+05	6,11E+05	10	2,73E+05	1,31E+05	2,62E+05	10	-0,5	0,3	-2,0	1,7	3
**tetrachloroethylene**	3,41E+05	1,54E+05	3,40E+05	10	9,13E+04	2,93E+04	8,29E+04	10	-0,7	0,7	-2,5	1,5	4
**tetradecane**	5,17E+05	2,95E+05	4,46E+05	10	9,72E+04	7,44E+04	8,28E+04	10	-0,04	0,3	-0,4	0,9	5
**toluene**	2,71E+06	2,23E+06	2,09E+06	10	8,91E+05	3,90E+05	7,99E+05	10	-1,3	1,2	-4,6	2,8	3
**trichloromethane**	4,84E+04	2,58E+04	4,11E+04	10	2,11E+04	7,63E+03	1,89E+04	10	-5,3	2,3	-44	58	2
**undecane, 5-methyl**	1,33E+05	2,91E+05	/	2	4,77E+04	1,01E+05	/	2	1,0	0,0	-0,1	1,6	3
**ion 43 - rt 7.343**	1,48E+06	9,96E+05	1,20E+06	10	8,16E+05	5,43E+05	7,02E+05	10	-0,3	0,8	0,1	0,4	2
**ion 43 - rt 5.888**	1,98E+06	2,61E+06	9,53E+05	10	6,17E+05	7,57E+05	2,46E+05	10	-1,6	2,3	-4,2	4,7	3

Table 4. Mean, Median and Standard Deviation (STD.DEV) of peak area of the 103 VOCs detected in breath samples collected using Tedlar Bags and Mistral. Number (N) of breath samples in which VOC were detected is also reported in addition to the ratio between peak area of VOC detected in samples collected from 10 healthy subjects using Mistral and Bag and to p-value of the comparison.

Compound in HC Breath Sample	Tedlar Bag			N	Mistral			N	Mistral/Tedlar Bag	p-value
	Mean	STD.DEV	Median		Mean	STD.DEV	Median			
1,3-cyclopentadiene	2.58E+04	3.06E+04	1,33E+04	10	2.26E+04	1.81E+04	2,47E+04	9	0,88	0,33
1-hexanol, 2-ethyl-	1.06E+06	8.95E+05	7,01E+05	10	2.14E+06	1.27E+06	2,15E+06	10	2,02	0,16
1-octene	3.13E+05	3.33E+05	2,10E+05	10	2.77E+05	1.81E+05	2,96E+05	9	0,88	0,33
1-propanol	8.41E+05	6.80E+05	1,04E+06	9	5.88E+05	4.80E+05	5,36E+05	9	0,7	0,22
1-propanol, 2-butoxy-	7.84E+03	1.92E+04	/	2	2.05E+04	1.81E+04	2,26E+04	7	2,62	0,34
1-propene, 2methyl-	1.02E+06	1.06E+06	8,57E+05	8	6.56E+05	8.43E+05	3,83E+05	7	0,64	0,22
2,3-butandione	4.76E+05	7.28E+05	2,53E+05	10	2.40E+05	1.38E+05	2,08E+05	10	0,5	0,30
2,4-dimethyl heptane	1.13E+05	1.37E+05	6,03E+04	9	6.22E+03	1.34E+04	/	2	0,06	0,29
2-butanone	7.82E+05	1.57E+06	2,91E+05	10	4.99E+05	2.86E+05	4,00E+05	10	0,64	0,33
2-butanone, 3-hydroxy-	9.04E+04	2.26E+05	8,87E+03	5	8.08E+04	1.19E+05	/	4	0,89	0,35
2-methyl butane - ion 43	4.58E+05	6.58E+05	3,19E+05	8	2.89E+05	2.87E+05	2,34E+05	7	0,63	0,30
2-methyl butane - ion 57	3.46E+05	3.18E+05	3,51E+05	8	2.60E+05	1.94E+05	2,38E+05	8	0,75	0,31
2-methyl hexane	8.72E+04	1.68E+05	3,95E+04	9	3.33E+04	3.23E+04	3,72E+04	6	0,38	0,32
2-pentanone	5.46E+05	7.53E+05	2,82E+05	10	3.59E+05	4.95E+05	3,06E+05	8	0,66	0,25
3-carene	1.43E+04	2.20E+04	/	4	3.97E+04	1.15E+05	/	3	2,78	0,36
3-hexene	7.43E+03	1.50E+04	/	3	2.56E+03	5.52E+03	3,17E+02	5	0,34	0,33
3-methyl hexane	4.09E+04	9.02E+04	1,35E+04	7	4.37E+03	7.26E+03	/	3	0,11	0,32
4-carene	3.97E+04	3.80E+04	2,12E+04	10	1.94E+04	8.83E+03	1,88E+04	10	0,49	0,32
4-methyl octane	1.75E+05	8.80E+04	1,41E+05	10	5.40E+04	1.22E+05	/	3	0,31	0,27
acetaldehyde - ion 44	3.18E+06	3.70E+06	1,78E+06	9	2.35E+06	2.06E+06	1,49E+06	10	0,74	0,19
acetaldehyde - ion 43	1.86E+06	2.10E+06	1,00E+06	9	1.45E+06	1.19E+06	1,01E+06	10	0,78	0,27
acetone	9.25E+06	4.04E+06	7,67E+06	10	9.56E+06	5.88E+06	8,09E+06	10	1,03	0,15
acetophenone	6.38E+06	5.42E+06	5,79E+06	10	8.43E+06	6.94E+06	7,78E+06	10	1,32	0,76
acid acetic methylester - ion 43	3.64E+05	2.93E+05	2,87E+05	10	2.43E+05	1.20E+05	2,59E+05	9	0,67	0,29
acid acetic methylester - ion 74	1.13E+05	6.98E+04	1,08E+05	9	8.34E+04	4.30E+04	8,33E+04	9	0,74	0,31
alpha-pinene	1.39E+05	3.96E+04	1,43E+05	10	1.33E+05	5.90E+04	1,45E+05	10	0,96	0,32
benzaldehyde	3.83E+06	3.05E+06	3,24E+06	10	5.07E+06	3.17E+06	4,81E+06	10	1,32	0,89
benzene - ion 78	8.54E+06	1.04E+07	3,78E+06	10	6.62E+06	7.87E+06	2,76E+06	10	0,77	0,04
benzene - ion 52	1.21E+06	1.46E+06	5,74E+05	10	9.50E+05	1.09E+06	4,06E+05	10	0,78	0,27
benzene, tetramethyl-	8.83E+04	2.75E+04	8,44E+04	10	9.54E+04	3.50E+04	9,49E+04	10	1,08	0,33
benzoic acid	3.18E+07	2.64E+07	2,73E+07	10	3.87E+07	2.96E+07	3,72E+07	10	1,22	0,0003

**benzonitrile**	2.62E+06	2.16E+06	2,92E+06	10	4.51E+06	4.07E+06	3,98E+06	10	1,72	0,97
**bromobenzene**	6.67E+04	5.90E+04	7,14E+04	7	9.71E+04	9.33E+04	1,07E+05	6	1,46	0,34
**butanal - ion 41**	2.16E+04	3.42E+04	5,22E+03	8	3.77E+03	4.28E+03	2,61E+03	5	0,18	0,33
**butanal - ion 72**	2.30E+05	4.32E+05	8,70E+04	10	1.60E+05	9.49E+04	1,28E+05	10	0,7	0,33
**chlorobenzene**	4.56E+05	6.51E+05	2,94E+05	10	4.86E+05	4.14E+05	5,21E+05	10	1,07	0,32
**chloromethane**	1.66E+05	1.15E+05	1,28E+05	10	1.14E+05	9.61E+04	7,26E+04	9	0,69	0,31
**ciclopropane, 1,1-dimethyl-2-(3methyl-1i**	6.80E+04	2.36E+04	6,43E+04	10	4.44E+04	2.33E+04	4,18E+04	10	0,65	0,32
**cyclohexane**	2.83E+04	3.76E+04	1,67E+04	10	1.24E+04	6.87E+03	1,33E+04	9	0,44	0,33
**cyclohexane, methyl- - ion 55**	6.97E+04	9.19E+04	4,29E+04	9	4.00E+04	3.26E+04	4,62E+04	7	0,57	0,33
**cyclohexane, methyl- - ion 83**	3.94E+04	3.23E+04	3,09E+04	9	2.89E+04	2.48E+04	3,15E+04	7	0,73	0,33
**cyclohexanone**	1.46E+05	1.40E+05	1,08E+05	10	2.65E+05	1.67E+05	2,55E+05	10	1,81	0,38
**cyclopentane - ion 42**	1.49E+06	4.08E+06	/	4	1.01E+05	2.95E+05	/	3	0,07	0,15
**cyclopentane - ion 70**	5.17E+05	1.60E+06	7,63E+03	7	8.04E+03	9.22E+03	5,24E+03	5	0,02	0,26
**cyclopentene, dimethyl-**	5.79E+06	4.42E+06	4,47E+06	9	6.15E+06	5.17E+06	4,89E+06	10	1,06	0,08
**decanal**	5.51E+06	2.98E+06	5,28E+06	10	2.69E+06	1.30E+06	2,48E+06	10	0,49	0,0005
**decane**	1.68E+05	1.09E+05	1,44E+05	10	1.96E+05	9.85E+04	2,30E+05	10	1,17	0,35
**decane, 3,7-dimethyl-**	6.10E+05	1.91E+05	6,16E+05	10	1.59E+05	8.20E+04	1,74E+05	10	0,26	0,15
**decane, dimethyl-**	2.13E+05	8.18E+04	3,13E+05	10	1.33E+04	2.25E+04	/	3	0,06	0,24
**dimethyl disulphide**	1.77E+05	2.69E+05	4,61E+04	10	1.89E+05	3.14E+05	6,78E+04	9	1,06	0,32
**dimethyl sulphide**	4.27E+05	4.54E+05	2,64E+05	10	3.78E+05	2.90E+05	2,78E+05	10	0,88	0,25
**ethanol - ion 45**	8.95E+06	7.75E+06	7,96E+06	10	7.16E+06	4.25E+06	6,57E+06	10	0,8	0,16
**ethanol - ion 46**	3.41E+06	3.19E+06	2,86E+06	10	2.51E+06	1.48E+06	2,25E+06	10	0,74	0,21
**ethanol - ion 43**	1.88E+06	1.55E+06	1,52E+06	10	1.37E+06	9.10E+05	1,14E+06	10	0,73	0,22
**ethyl acetate**	1.95E+05	3.03E+05	8,86E+04	10	7.13E+04	4.32E+04	8,05E+04	9	0,37	0,30
**ethylbenzene**	2.02E+05	1.13E+05	1,96E+05	10	5.49E+05	2.22E+05	5,41E+05	10	2,72	0,51
**heptanal**	4.85E+05	8.13E+05	2,41E+05	10	3.17E+05	1.64E+05	2,96E+05	10	0,65	0,31
**heptane**	7.78E+04	1.58E+05	3,62E+04	8	2.68E+04	2.89E+04	2,14E+04	6	0,34	0,33
**hexanal - ion 44**	4.49E+05	7.86E+05	1,97E+05	10	5.60E+05	2.74E+05	4,76E+05	10	1,25	0,44
**hexanal - ion 56**	4.51E+05	7.69E+05	2,10E+05	10	6.85E+05	3.24E+05	5,91E+05	10	1,52	0,51
**hexane - ion 57**	8.18E+05	7.74E+05	4,79E+05	10	2.22E+05	1.63E+05	2,03E+05	9	0,27	0,15
**hexane - ion 86**	1.38E+05	1.33E+05	7,78E+04	10	3.79E+04	2.43E+04	3,35E+04	10	0,27	0,29
**ion 46 - rt 15.464**	/	/	/	0	1.20E+02	3.79E+02	/	1	/	/
**ion 56 - rt 9.401**	3.69E+05	4.07E+05	2,37E+05	10	3.37E+05	1.95E+05	3,01E+05	10	0,91	0,35
**ion 57 - rt 26.465**	9.58E+03	1.08E+04	6,01E+03	5	1.05E+05	6.52E+04	1,38E+05	10	10,99	0,37
**ion 57 - rt 26.488**	6.54E+03	8.91E+03	/	4	1.03E+05	6.60E+04	1,26E+05	9	15,7	0,37
**ion 57 - rt 25.056**	2.64E+05	7.59E+04	2,55E+05	10	5.71E+05	3.19E+05	6,74E+05	10	2,16	0,46
**ion 57 - rt 36.442**	1.99E+05	9.55E+04	1,90E+05	10	8.70E+05	5.77E+05	1,11E+06	9	4,37	0,66

**ion 79 - rt 10.315**	2.07E+07	1.84E+07	1,39E+07	10	1.91E+07	1.43E+07	1,47E+07	10	0,92	0,02
**isobutane**	1.72E+05	3.22E+05	/	3	1.14E+05	1.50E+05	/	4	0,66	0,33
**isoprene**	1.28E+07	5.97E+06	1,37E+07	10	1.49E+07	3.72E+06	1,48E+07	10	1,16	0,19
**isopropanol**	2.88E+06	1.58E+06	2,86E+06	10	2.70E+06	1.88E+06	2,07E+06	10	0,94	0,17
**isopropyl toluene**	1.01E+05	6.58E+04	7,17E+04	10	1.04E+05	5.56E+04	1,12E+05	10	1,03	0,33
**m/p-xilene**	5.85E+05	4.23E+05	5,24E+05	10	1.06E+06	4.11E+05	1,07E+06	10	1,81	0,63
**methyl ciclopentane**	5.59E+04	6.75E+04	3,01E+04	10	3.50E+04	2.61E+04	2,87E+04	9	0,63	0,33
**methyl vinyl ketone**	4.53E+05	6.44E+05	2,42E+05	10	4.06E+05	2.59E+05	3,56E+05	10	0,9	0,35
**methylene chloride**	4.79E+06	1.48E+07	4,77E+04	9	5.81E+04	5.08E+04	3,74E+04	10	0,01	0,02
**naphtalene**	2.70E+05	1.48E+05	2,28E+05	10	3.86E+05	1.84E+05	3,50E+05	10	1,43	0,38
**nonanal**	4.11E+06	2.80E+06	3,56E+06	10	3.32E+06	1.68E+06	3,35E+06	10	0,81	0,08
**nonane**	3.94E+05	3.55E+05	3,02E+05	10	2.45E+05	1.77E+05	2,17E+05	10	0,62	0,27
**octanal - ion 43**	1.44E+06	1.44E+06	1,03E+06	10	6.82E+05	3.46E+05	6,87E+05	10	0,47	0,12
**octanal - ion 57**	1.21E+06	1.16E+06	8,87E+05	10	5.93E+05	3.01E+05	6,11E+05	10	0,49	0,15
**octane**	3.11E+05	3.28E+05	2,04E+05	10	2.46E+05	1.80E+05	2,20E+05	9	0,79	0,31
**o-xilene**	1.59E+05	9.70E+04	1,37E+05	10	4.08E+05	1.81E+05	4,11E+05	10	2,56	0,46
**para-benzoquinone**	1.46E+05	1.35E+05	1,62E+05	7	9.99E+04	8.26E+04	1,16E+05	7	0,68	0,32
**pentanal**	4.04E+05	1.00E+06	9,74E+04	9	2.13E+05	2.02E+05	1,79E+05	7	0,53	0,32
**pentane - ion 43**	2.70E+05	1.91E+05	2,30E+05	9	3.14E+05	2.77E+05	2,47E+05	8	1,16	0,32
**pentane - ion 57**	6.99E+04	4.51E+04	7,52E+04	9	6.78E+04	5.41E+04	5,85E+04	8	0,97	0,33
**pentane, 2-methyl- - ion 43**	2.19E+05	1.33E+05	2,57E+05	9	1.90E+05	1.58E+05	1,77E+05	8	0,87	0,31
**pentane, 2-methyl- - ion 71**	1.44E+05	8.53E+04	1,52E+05	9	1.32E+05	6.31E+04	1,35E+05	10	0,92	0,33
**phenylethyne**	1.74E+06	1.51E+06	1,79E+06	10	2.32E+06	1.80E+06	2,24E+06	10	1,33	0,58
**ion 57 - rt 14.989**	5.54E+03	9.82E+03	/	4	2.35E+03	3.88E+03	/	3	0,42	0,33
**propanoic acid**	5.22E+05	1.31E+06	2,30E+04	6	7.57E+04	9.97E+04	1,97E+04	5	0,14	0,27
**propylbenzene**	7.22E+04	4.72E+04	5,67E+04	10	1.25E+05	6.51E+04	1,36E+05	10	1,73	0,36
**sevoflurane**	8.85E+04	1.11E+05	7,14E+04	8	1.78E+05	1.31E+05	1,14E+05	10	2,01	0,37
**styrene**	2.13E+05	1.44E+05	1,78E+05	10	5.13E+05	2.87E+05	4,43E+05	10	2,4	0,46
**tetrachloroethylene**	1.25E+05	5.35E+04	1,10E+05	10	2.08E+05	7.50E+04	1,94E+05	10	1,66	0,37
**tetradecane**	2.32E+05	2.33E+05	1,63E+05	10	4.90E+05	2.17E+05	4,50E+05	10	2,11	0,47
**toluene**	1.15E+06	7.30E+05	1,04E+06	10	1.14E+06	4.97E+05	1,27E+06	10	0,99	0,37
**trichloromethane**	1.34E+05	3.31E+05	3,04E+04	10	6.55E+03	4.42E+03	6,26E+03	9	0,05	0,31
**undecane, 5-methyl-**	1.33E+05	4.20E+05	/	1	2.34E+05	5.04E+05	/	2	1,76	0,32
**ion 43 - rt 7.343**	1.60E+06	1.67E+06	9,82E+05	9	1.12E+06	9.03E+05	7,64E+05	10	0,7	0,33
**ion 43 - rt 5.888**	2.27E+06	1.84E+06	1,86E+06	10	1.81E+06	1.90E+06	1,05E+06	10	0,8	0,14

Table 5. Mean, Median and Standard Deviation (STD.DEV) of peak area of the 103 VOCs detected in breath samples collected using RecCIVA Low and ReCIVA Whole. Number (N) of breath samples in which VOC were detected is also reported in addition to the ratio between peak area of VOC detected in samples collected from 10 healthy subjects by using RecCIVA Low and ReCIVA Whole and to p-value of the comparison.

Compound in HC Breath Sample	ReCIVA Low			N	ReCIVA Whole			N	ReCIVA Low/ReCIVA Whole	p-value
	Mean	STD.DEV	Median		Mean	STD.DEV	Median			
1,3-cyclopentadiene	1.30E+04	1.42E+04	9,44E+03	7	2.19E+04	2.31E+04	1,41E+04	9	0,59	0,99
1-hexanol, 2-ethyl-	1.00E+05	4.08E+04	9,04E+04	10	1.34E+05	1.02E+05	8,76E+04	10	0,75	0,74
1-octene	3.65E+04	5.52E+04	2,24E+04	6	1.90E+05	2.38E+05	9,07E+04	10	0,19	0,85
1-propanol	1.21E+04	3.81E+04	/	1	7.53E+04	5.42E+04	8,76E+04	8	0,16	0,84
1-propanol, 2-butoxy-	/	/	/	/	/	/	/	/	/	/
1-propene, 2methyl-	1.24E+05	1.47E+05	6,72E+04	7	3.33E+05	4.45E+05	1,46E+05	6	0,37	0,96
2,3-butandione	9.09E+04	8.10E+04	8,47E+04	8	3.04E+05	2.54E+05	1,66E+05	10	0,3	0,56
2,4-dimethyl heptane	1.67E+03	5.29E+03	/	1	1.87E+04	2.15E+04	9,68E+03	7	0,09	0,97
2-butanone	8.72E+04	6.09E+04	7,95E+04	9	4.43E+05	3.58E+05	3,40E+05	10	0,20	0,48
2-butanone, 3-hydroxy-	2.22E+05	2.08E+05	2,22E+05	7	4.81E+05	1.88E+05	4,16E+05	10	0,46	0,33
2-methyl butane - ion 43	5.40E+04	9.64E+04	0,00E+00	4	1.11E+05	1.30E+05	4,92E+04	7	0,49	0,90
2-methyl butane - ion 57	4.24E+04	6.57E+04	1,86E+04	5	1.04E+05	1.43E+05	4,19E+04	7	0,41	0,99
2-methyl hexane	/	/	/	0	4.66E+04	7.54E+04	5,96E+03	5	/	/
2-pentanone	2.74E+04	6.15E+04	/	3	2.63E+05	2.66E+05	1,99E+05	8	0,10	0,68
3-carene	4.56E+03	7.78E+03	/	3	9.47E+02	2.99E+03	/	1	4,82	0,97
3-hexene	/	/	/	/	8.88E+03	1.49E+04	5,08E+02	6	/	/
3-methyl hexane	/	/	/	/	2.20E+04	5.01E+04	/	3	/	/
4-carene	1.85E+04	2.07E+04	9,57E+03	10	1.36E+04	1.47E+04	9,62E+03	9	1,36	0,98
4-methyl octane	2.15E+04	1.85E+04	2,29E+04	7	4.03E+04	3.73E+04	2,45E+04	10	0,53	0,98
acetaldehyde - ion 44	1.37E+06	1.72E+06	1,26E+06	7	5.74E+05	4.75E+05	4,63E+05	9	2,38	0,07
acetaldehyde - ion 43	7.46E+05	9.32E+05	6,57E+05	7	4.22E+05	3.43E+05	3,26E+05	9	1,77	0,45
acetone	3.40E+06	6.82E+05	3,61E+06	10	2.43E+06	1.26E+06	2,32E+06	10	1,40	0,00001
acetophenone	5.12E+06	5.33E+06	2,97E+06	10	8.63E+06	8.81E+06	5,48E+06	10	0,59	0,29
acid acetic methylester - ion 43	4.19E+04	4.65E+04	3,12E+04	6	1.06E+05	8.65E+04	8,50E+04	10	0,4	0,92
acid acetic methylester - ion 74	1.56E+04	1.72E+04	1,10E+04	6	4.18E+04	2.74E+04	3,97E+04	10	0,37	0,97
alpha-pinene	3.92E+04	2.23E+04	4,18E+04	10	4.65E+04	3.07E+04	3,71E+04	10	0,84	0,98
benzaldehyde	2.68E+06	2.09E+06	1,94E+06	10	4.03E+06	3.46E+06	2,99E+06	10	0,66	0,79
benzene - ion 78	1.78E+06	1.60E+06	1,26E+06	10	1.75E+07	1.49E+07	1,19E+07	10	0,10	0,00002
benzene - ion 52	2.65E+05	2.40E+05	1,89E+05	10	2.84E+06	2.77E+06	1,74E+06	10	0,09	0,00001
benzene, tetramethyl-	1.39E+04	7.02E+03	1,08E+04	10	2.25E+04	1.65E+04	1,76E+04	10	0,62	1,00
benzoic acid	2.65E+07	2.83E+07	1,61E+07	10	3.50E+07	3.44E+07	2,50E+07	10	0,76	0,00001

benzonitrile	2.10E+06	2.12E+06	1,48E+06	10	3.83E+06	4.54E+06	1,94E+06	10	0,55	0,75
bromobenzene	4.54E+04	5.70E+04	3,08E+04	6	1.14E+05	1.42E+05	6,42E+04	6	0,40	0,98
butanal - ion 41	1.04E+03	1.74E+03	/	3	2.29E+03	5.12E+03	/	3	0,46	0,98
butanal - ion 72	2.70E+04	1.87E+04	2,61E+04	9	1.24E+05	9.64E+04	9,13E+04	10	0,22	0,85
chlorobenzene	1.86E+05	2.11E+05	8,80E+04	10	1.25E+06	2.62E+06	2,94E+05	10	0,15	0,46
chloromethane	2.24E+04	2.38E+04	1,93E+04	5	1.45E+05	2.35E+05	6,41E+04	10	0,15	0,86
ciclopropane, 1,1-dimethyl-2-(3methyl-1i	2.38E+04	1.03E+04	2,14E+04	10	2.44E+04	8.18E+03	2,21E+04	10	0,98	0,98
cyclohexane	1.94E+03	3.43E+03	/	3	3.35E+04	3.99E+04	1,77E+04	9	0,06	0,97
cyclohexane, methyl- - ion 55	8.00E+03	1.29E+04	0,00E+00	4	2.67E+04	1.88E+04	2,67E+04	9	0,30	0,98
cyclohexane, methyl- - ion 83	5.47E+03	1.04E+04	0,00E+00	4	1.35E+04	1.68E+04	4,05E+03	9	0,40	0,98
cyclohexanone	5.97E+04	3.59E+04	4,94E+04	10	1.01E+05	6.51E+04	9,18E+04	10	0,59	0,96
cyclopentane - ion 42	1.60E+05	1.06E+05	1,78E+05	8	1.62E+05	1.02E+05	1,53E+05	9	0,99	0,94
cyclopentane - ion 70	3.31E+04	2.09E+04	3,90E+04	8	3.71E+04	2.29E+04	3,20E+04	9	0,89	1,00
cyclopentene, dimethyl-	3.52E+06	3.44E+06	2,68E+06	9	4.00E+06	4.47E+06	2,03E+06	10	0,88	0,05
decanal	4.26E+05	2.43E+05	2,98E+05	10	1.17E+06	2.17E+06	5,09E+05	10	0,36	0,61
decane	1.04E+05	4.94E+04	1,21E+05	10	1.55E+05	1.19E+05	1,35E+05	10	0,67	0,98
decane, 3,7-dimethyl-	7.78E+04	3.49E+04	7,13E+04	10	1.31E+05	1.01E+05	9,11E+04	10	0,59	0,97
decane, dimethyl-	1.50E+04	8.05E+03	7,49E+04	9	2.55E+04	2.15E+04	7,66E+04	9	0,59	0,99
dimethyl disulphide	1.91E+04	1.65E+04	1,13E+04	10	4.05E+04	6.15E+04	1,69E+04	10	0,47	0,99
dimethyl sulphide	1.51E+05	1.83E+05	9,14E+04	8	1.20E+05	1.22E+05	7,23E+04	10	1,26	0,92
ethanol - ion 45	9.87E+05	9.80E+05	6,03E+05	9	6.50E+05	4.78E+05	5,23E+05	10	1,52	0,47
ethanol - ion 46	3.62E+05	3.49E+05	2,28E+05	9	2.31E+05	1.70E+05	1,80E+05	10	1,57	0,78
ethanol - ion 43	3.38E+05	3.47E+05	2,62E+05	9	1.04E+05	1.09E+05	6,36E+04	10	3,25	0,55
ethyl acetate	2.80E+05	3.15E+05	9,54E+04	9	7.62E+05	8.72E+05	4,54E+05	9	0,37	0,16
ethylbenzene	4.81E+04	2.41E+04	5,12E+04	10	1.00E+05	8.65E+04	7,59E+04	10	0,48	0,92
heptanal	4.49E+04	2.40E+04	3,47E+04	10	1.24E+05	1.56E+05	7,14E+04	10	0,36	0,89
heptane	6.10E+03	8.71E+03	2,39E+03	6	2.25E+05	4.17E+05	3,26E+04	8	0,03	0,36
hexanal - ion 44	4.61E+04	2.05E+04	4,15E+04	10	1.35E+05	1.79E+05	8,44E+04	10	0,34	0,89
hexanal - ion 56	4.13E+04	2.43E+04	3,38E+04	10	1.38E+05	1.91E+05	7,41E+04	10	0,30	0,88
hexane - ion 57	3.19E+04	4.12E+04	1,53E+04	6	3.39E+05	7.00E+05	5,44E+04	9	0,09	0,84
hexane - ion 86	7.88E+03	8.30E+03	5,24E+03	7	6.60E+04	1.37E+05	1,03E+04	8	0,12	0,99
ion 46 - rt 15.464	5.62E+01	1.78E+02	/	1	4.75E+04	1.50E+05	/	1	1,18	0,82
ion 56 - rt 9.401	1.07E+05	7.68E+04	9,68E+04	9	3.19E+05	3.08E+05	2,05E+05	10	0,34	0,74
ion 57 - rt 26.465	9.50E+04	4.69E+04	9,27E+04	10	1.08E+05	6.51E+04	1,07E+05	9	0,88	0,93
ion 57 - rt 26.488	9.37E+04	4.90E+04	9,15E+04	10	1.20E+05	5.43E+04	1,13E+05	10	0,78	0,93
ion 57 - rt 25.056	6.17E+05	3.42E+05	6,12E+05	10	7.92E+05	3.80E+05	8,04E+05	10	0,78	0,58

ion 57 - rt 36.442	3.42E+04	4.39E+04	9,51E+03	5	3.45E+04	5.09E+04	0,00E+00	4	0,99	0,96
ion 79 - rt 10.315	1.11E+07	7.22E+06	1,04E+07	10	1.39E+07	1.54E+07	8,15E+06	10	0,80	0,00001
isobutane	7.40E+03	2.34E+04	/	1	7.00E+03	2.21E+04	/	1	1,06	0,99
isoprene	3.98E+06	1.67E+06	3,47E+06	10	3.04E+06	6.51E+05	3,07E+06	10	1,31	0,04
isopropanol	3.02E+05	2.22E+05	3,55E+05	9	3.17E+05	1.80E+05	3,24E+05	10	0,95	0,98
isopropyl toluene	3.67E+04	2.57E+04	2,45E+04	10	5.03E+04	4.61E+04	2,53E+04	10	0,73	0,99
m/p-xilene	1.03E+05	6.13E+04	7,13E+04	10	2.06E+05	2.10E+05	1,27E+05	10	0,50	0,92
methyl ciclopentane	3.49E+03	6.05E+03	/	3	3.26E+04	4.18E+04	2,56E+04	7	0,11	0,98
methyl vinyl ketone	7.11E+04	4.46E+04	5,84E+04	10	6.05E+05	6.04E+05	4,01E+05	10	0,12	0,42
methylene chloride	9.52E+04	7.42E+04	1,16E+05	7	1.92E+05	1.98E+05	9,74E+04	10	0,50	0,91
naphtalene	1.17E+05	7.68E+04	1,00E+05	10	2.04E+05	1.75E+05	1,31E+05	10	0,57	0,96
nonanal	2.75E+05	1.32E+05	2,16E+05	10	8.69E+05	1.61E+06	3,73E+05	10	0,32	0,56
nonane	5.58E+04	4.64E+04	3,63E+04	10	2.19E+05	3.08E+05	8,16E+04	10	0,26	0,88
octanal - ion 43	1.33E+05	6.74E+04	1,04E+05	10	3.62E+05	5.21E+05	2,08E+05	10	0,37	0,79
octanal - ion 57	1.29E+05	8.27E+04	9,22E+04	10	3.24E+05	4.47E+05	1,99E+05	10	0,40	0,84
octane	3.49E+04	5.54E+04	1,41E+04	5	1.89E+05	2.34E+05	9,14E+04	10	0,19	0,85
o-xilene	2.21E+04	1.17E+04	1,98E+04	10	5.48E+04	6.37E+04	3,07E+04	10	0,40	0,98
para-benzoquinone	2.78E+04	4.91E+04	1,12E+04	7	3.64E+05	4.71E+05	1,47E+05	8	0,08	0,77
pentanal	1.28E+04	2.07E+04	/	3	1.08E+05	9.52E+04	7,55E+04	10	0,12	0,81
pentane - ion 43	4.29E+04	6.86E+04	1,55E+04	5	9.76E+04	1.21E+05	5,58E+04	9	0,44	0,98
pentane - ion 57	9.97E+03	1.61E+04	3,82E+03	5	2.52E+04	2.98E+04	1,46E+04	9	0,40	1,00
pentane, 2-methyl- - ion 43	2.21E+04	3.58E+04	0,00E+00	4	6.24E+04	1.01E+05	1,74E+04	6	0,35	0,99
pentane, 2-methyl- - ion 71	1.20E+04	1.60E+04	5,93E+03	5	4.32E+04	5.76E+04	2,04E+04	7	0,28	1,00
phenylethyne	7.97E+05	6.72E+05	5,15E+05	10	2.93E+06	3.00E+06	2,44E+06	10	0,27	0,03
ion 57 - rt 14.989	1.49E+03	4.72E+03	/	1	1.89E+04	2.62E+04	5,06E+03	5	0,08	0,98
propanoic acid	/	/	/	/	1.81E+05	2.48E+05	0,00E+00	4	/	/
propylbenzene	1.79E+04	1.27E+04	1,47E+04	9	3.84E+04	3.75E+04	2,40E+04	10	0,47	0,99
sevoflurane	1.49E+04	1.68E+04	1,12E+04	6	1.75E+04	1.65E+04	1,36E+04	7	0,85	0,98
styrene	1.25E+05	1.01E+05	7,96E+04	10	2.32E+05	2.01E+05	1,56E+05	10	0,54	0,92
tetrachloroethylene	3.08E+04	1.75E+04	2,77E+04	10	9.77E+04	1.47E+05	3,62E+04	10	0,32	0,97
tetradecane	8.81E+04	6.44E+04	6,67E+04	10	1.28E+05	1.10E+05	8,03E+04	10	0,69	1,00
toluene	1.97E+05	1.35E+05	1,35E+05	10	1.02E+06	1.53E+06	2,69E+05	10	0,19	0,41
trichloromethane	5.11E+02	1.55E+03	/	2	1.84E+04	4.16E+04	4,42E+03	8	0,03	0,99
undecane, 5-methyl-	1.90E+04	4.03E+04	/	2	4.74E+04	1.19E+05	/	2	0,40	1,00
ion 43 - rt 7.343	6.12E+05	5.69E+05	5,59E+05	7	7.12E+05	6.82E+05	3,97E+05	10	0,86	0,55
ion 43 - rt 5.888	1.69E+05	1.89E+05	9,98E+04	9	5.44E+05	6.09E+05	3,21E+05	9	0,31	0,30

Table 6. Peak area of the 103 VOCs detected in breath samples collected using Tedlar Bags, Mistral, ReCIVA Low and ReCIVA Whole from 1 subject affected by lung cancer (LC) and in Ambient Air samples collected using Mistral and ReCIVA.

Compound in LC Breath Sample	Ambient Air		LC Breath Sample			
	ReCIVA	Mistral	Tedlar Bag	Mistral	ReCIVA Low	ReCIVA Whole
**1,3-cyclopentadiene**	5.84E+03	6.22E+03	3.31E+03	/	/	1.08E+04
**1-hexanol, 2-ethyl-**	1.01E+05	8.80E+05	7.36E+05	5.73E+05	7.43E+04	1.34E+05
**1-octene**	1.23E+05	9.65E+04	9.81E+04	/	/	1.08E+05
**1-propanol**	/	2.84E+04	/	8.86E+04	/	/
**1-propanol, 2-butoxy-**	/	/	/	/	/	/
**1-propene, 2-methyl-**	/	/	3.15E+05	1.46E+05	/	/
**2,3-butandione**	1.68E+05	2.38E+05	2.34E+05	1.30E+05	/	3.17E+05
**2,4-dimethyl heptane**	1.36E+05	2.05E+04	1.57E+05	/	/	6.25E+04
**2-butanone**	1.88E+06	2.39E+06	3.41E+05	3.06E+05	/	5.32E+05
**2-butanone, 3-hydroxy-**	2.79E+05	5.75E+04	1.77E+04	/	/	8.19E+05
**2-methyl butane - ion 43**	7.10E+04	1.21E+05	/	/	/	/
**2-methyl butane - ion 57**	8.25E+04	1.35E+05	/	/	/	/
**2-methyl hexane**	/	1.15E+05	9.36E+03	/	/	/
**2-pentanone**	1.48E+05	1.08E+05	2.76E+05	3.51E+04	1.70E+04	2.42E+05
**3-carene**	/	9.97E+03	/	/	/	/
**3-hexene**	2.96E+03	4.60E+03	/	/	/	/
**3-methyl hexane**	/	5.95E+04	1.13E+04	/	/	/
**4-carene**	/	1.40E+04	1.75E+04	5.59E+03	7.08E+03	7.12E+03
**4-methyl octane**	1.56E+05	/	2.52E+05	/	2.56E+04	7.69E+04
**acetaldehyde - ion 44**	1.89E+06	7.99E+06	1.49E+06	1.06E+06	/	1.59E+05
**acetaldehyde - ion 43**	1.90E+06	4.46E+06	8.73E+05	5.64E+05	/	1.30E+05
**acetone**	2.79E+05	1.28E+06	6.80E+06	6.64E+06	2.97E+06	1.46E+06
**acetophenone**	1.35E+06	1.01E+06	9.37E+05	1.44E+06	1.36E+06	1.49E+06
**acid acetic methylester - ion 43**	/	1.87E+05	3.32E+05	2.91E+05	/	5.62E+04
**acid acetic methylester - ion 74**	/	5.26E+04	1.22E+05	/	/	2.16E+04
**alpha-pinene**	2.77E+04	8.30E+04	7.22E+04	3.76E+04	1.33E+04	1.35E+04
**benzaldehyde**	1.47E+06	1.43E+06	8.19E+05	1.71E+06	1.13E+06	1.50E+06
**benzene - ion 78**	5.94E+06	5.96E+06	1.64E+06	7.90E+05	4.51E+05	5.49E+06
**benzene - ion 52**	8.82E+05	8.78E+05	2.13E+05	1.16E+05	6.83E+04	8.01E+05
**benzene, tetramethyl-**	1.62E+04	4.33E+04	6.31E+04	4.66E+04	8.90E+03	2.74E+04
**benzoic acid**	3.60E+06	3.31E+06	2.43E+06	6.53E+06	5.04E+06	4.55E+06
**benzonitrile**	1.88E+05	2.48E+05	2.23E+05	3.94E+05	2.78E+05	3.31E+05

**bromobenzene**	4.48E+04	/	/	/	/	3.10E+04
**butanal - ion 41**	/	5.16E+04	/	/	/	/
**butanal - ion 72**	5.05E+05	6.83E+05	1.06E+05	1.01E+05	/	1.75E+05
**chlorobenzene**	4.94E+04	3.12E+04	2.67E+04	2.99E+04	2.71E+04	5.48E+04
**chloromethane**	4.87E+04	2.71E+04	1.33E+05	7.80E+04	/	3.87E+04
**ciclopropane, 1,1-dimethyl-2-(3methyl-1i**	5.26E+03	2.59E+04	4.89E+04	1.43E+04	1.27E+04	1.69E+04
**cyclohexane**	2.32E+04	4.39E+04	2.26E+04	2.10E+03	9.84E+03	4.58E+04
**cyclohexane, methyl- - ion 55**	1.68E+04	3.18E+04	3.26E+04	/	/	/
**cyclohexane, methyl- - ion 83**	1.56E+04	2.22E+04	1.40E+04	/	/	/
**cyclohexanone**	7.15E+04	8.36E+04	8.11E+04	6.24E+04	2.77E+04	1.10E+05
**cyclopentane - ion 42**	/	/	1.69E+04	/	/	3.70E+05
**cyclopentane - ion 70**	/	/	6.06E+03	2.15E+04	/	8.12E+04
**cyclopentene, dimethyl-**	6.52E+06	9.99E+05	4.28E+06	4.91E+05	/	7.70E+05
**decanal**	3.42E+05	1.20E+06	2.40E+06	1.01E+06	2.55E+05	4.11E+05
**decane**	2.48E+05	1.33E+05	6.64E+04	4.97E+04	1.43E+05	1.70E+05
**decane, 3,7-dimethyl-**	1.31E+05	7.96E+04	5.94E+05	4.78E+04	3.96E+04	9.74E+04
**decane, dimethyl-**	2.92E+04	1.93E+03	2.32E+05	/	1.18E+04	/
**dimethyl disulphide**	/	6.17E+03	1.80E+05	2.15E+05	1.19E+04	4.17E+04
**dimethyl sulphide**	1.27E+04	2.96E+04	4.09E+05	4.13E+05	/	1.03E+05
**ethanol - ion 45**	1.19E+07	2.69E+07	2.37E+06	3.17E+06	/	2.21E+05
**ethanol - ion 46**	4.44E+06	1.01E+07	8.49E+05	1.23E+06	/	8.64E+04
**ethanol - ion 43**	2.36E+06	5.15E+06	5.42E+05	5.11E+05	/	7.10E+04
**ethyl acetate**	/	3.12E+05	1.91E+05	8.22E+04	/	/
**ethylbenzene**	5.12E+05	5.08E+05	1.15E+05	2.59E+05	4.61E+04	1.80E+05
**heptanal**	1.34E+05	2.32E+05	1.64E+05	1.21E+05	1.85E+04	1.54E+05
**heptane**	5.88E+04	5.00E+04	1.26E+04	1.93E+04	/	1.31E+06
**hexanal - ion 44**	1.58E+05	6.39E+05	1.54E+05	2.97E+05	2.27E+04	1.30E+05
**hexanal - ion 56**	1.61E+05	7.97E+05	1.40E+05	3.64E+05	1.40E+04	1.31E+05
**hexane - ion 57**	4.02E+04	7.80E+04	1.69E+05	4.29E+04	/	2.10E+04
**hexane - ion 86**	7.93E+03	1.42E+04	3.48E+04	1.06E+04	8.20E+03	/
**ion 46 - rt 15.464**	/	9.76E+03	/	/	/	/
**ion 56 - rt 9.401**	2.04E+05	2.47E+05	1.28E+05	1.09E+05	1.87E+04	1.02E+05
**ion 57 - rt 26.465**	3.33E+05	6.27E+04	1.96E+04	3.53E+04	9.14E+04	2.34E+05
**ion 57 - rt 26.488**	3.40E+05	6.04E+04	2.22E+04	3.80E+04	9.33E+04	2.38E+05
**ion 57 - rt 25.056**	2.28E+06	3.75E+05	1.93E+05	1.81E+05	5.38E+05	1.54E+06
**ion 57 - rt 36.442**	1.18E+06	2.21E+05	3.11E+05	1.78E+05	1.06E+05	8.69E+04
**ion 79 - rt 10.315**	5.67E+06	9.18E+06	9.25E+06	8.65E+06	6.36E+06	6.80E+06

**isobutane**	/	/	/	/	/	/
**isoprene**	/	3.35E+05	9.82E+06	8.97E+06	3.38E+06	2.97E+06
**isopropanol**	2.00E+06	3.31E+06	1.06E+06	1.05E+06	/	1.69E+05
**isopropyl toluene**	3.13E+04	7.46E+04	4.86E+04	3.98E+04	1.73E+04	2.07E+04
**m/p-xilene**	3.41E+05	8.74E+05	3.41E+05	4.33E+05	5.41E+04	1.37E+05
**methyl ciclopentane**	2.37E+04	4.65E+04	1.14E+04	/	/	2.83E+04
**methyl vinyl ketone**	1.38E+05	1.91E+05	1.45E+05	1.02E+05	3.21E+04	2.96E+05
**methylene chloride**	4.11E+05	5.38E+04	3.36E+04	1.81E+05	/	2.18E+05
**naphtalene**	9.81E+04	2.15E+05	1.15E+05	1.60E+05	6.84E+04	1.07E+05
**nonanal**	4.64E+05	1.62E+06	2.18E+06	1.11E+06	1.51E+05	3.66E+05
**nonane**	1.06E+05	8.75E+04	1.21E+05	3.86E+04	1.72E+04	7.86E+04
**octanal - ion 43**	2.82E+05	3.71E+05	7.72E+05	2.21E+05	6.56E+04	2.75E+05
**octanal - ion 57**	2.15E+05	3.06E+05	6.84E+05	1.97E+05	5.96E+04	2.24E+05
**octane**	1.28E+05	9.91E+04	9.14E+04	/	/	1.10E+05
**o-xilene**	1.35E+05	3.70E+05	8.03E+04	1.52E+05	1.42E+04	4.19E+04
**para-benzoquinone**	7.83E+03	/	/	/	/	1.43E+04
**pentanal**	1.67E+05	1.86E+05	6.14E+04	/	/	1.88E+05
**pentane - ion 43**	/	/	1.33E+05	/	/	1.15E+04
**pentane - ion 57**	/	/	2.55E+04	/	/	4.57E+03
**pentane, 2-methyl- - ion 43**	/	1.80E+05	7.57E+04	/	/	/
**pentane, 2-methyl- - ion 71**	4.66E+04	9.86E+04	6.27E+04	4.63E+04	/	/
**phenylethyne**	3.59E+05	2.09E+05	9.62E+04	5.50E+05	2.90E+05	5.14E+05
**ion 57 - rt 14.989**	3.81E+05	1.42E+04	/	/	1.49E+04	7.41E+04
**propanoic acid**	3.58E+04	1.56E+05	/	/	/	/
**propylbenzene**	4.79E+04	7.33E+04	3.53E+04	3.12E+04	/	2.54E+04
**sevoflurane**	2.54E+04	7.21E+04	2.55E+04	2.06E+04	/	/
**styrene**	1.29E+05	3.56E+05	1.06E+05	1.93E+05	4.79E+04	9.60E+04
**tetrachloroethylene**	7.15E+04	3.51E+05	4.99E+04	1.34E+05	1.03E+04	1.46E+04
**tetradecane**	1.35E+04	2.59E+05	7.65E+04	1.94E+05	4.23E+04	8.27E+04
**toluene**	6.13E+05	1.07E+06	4.10E+05	4.02E+05	7.79E+04	2.61E+05
**trichloromethane**	3.75E+04	3.41E+04	1.81E+04	3.92E+03	/	/
**undecane, 5-methyl-**	/	/	/	/	/	/
**ion 43 - rt 7.343**	3.20E+05	6.57E+05	/	9.38E+05	/	3.15E+05
**ion 43 - rt 5.888**	1.35E+05	4.48E+05	3.22E+05	2.00E+05	/	3.88E+03
